# Chaos-Enhanced Adaptive Hybrid Butterfly Particle Swarm Optimization Algorithm for Passive Target Localization

**DOI:** 10.3390/s22155739

**Published:** 2022-07-31

**Authors:** Maja Rosić, Miloš Sedak, Mirjana Simić, Predrag Pejović

**Affiliations:** 1 Faculty of Mechanical Engineering, University of Belgrade, 11000 Belgrade, Serbia; msedak@mas.bg.ac.rs; 2 School of Electrical Engineering, University of Belgrade, 11000 Belgrade, Serbia; mira@etf.rs (M.S.); peja@etf.rs (P.P.)

**Keywords:** localization, time difference of arrival, butterfly optimization algorithm, hybrid optimization, particle swarm optimization, Cramer-Rao lower bound

## Abstract

This paper considers the problem of finding the position of a passive target using noisy time difference of arrival (TDOA) measurements, obtained from multiple transmitters and a single receiver. The maximum likelihood (ML) estimator’s objective function is extremely nonlinear and non-convex, making it impossible to use traditional optimization techniques. In this regard, this paper proposes the chaos-enhanced adaptive hybrid butterfly particle swarm optimization algorithm, named CAHBPSO, as the hybridization of butterfly optimization (BOA) and particle swarm optimization (PSO) algorithms, to estimate passive target position. In the proposed algorithm, an adaptive strategy is employed to update the sensory fragrance of BOA algorithm, and chaos theory is incorporated into the inertia weight of PSO algorithm. Furthermore, an adaptive switch probability is employed to combine global and local search phases of BOA with the PSO algorithm. Additionally, the semidefinite programming is employed to convert the considered problem into a convex one. The statistical comparison on CEC2014 benchmark problems shows that the proposed algorithm provides a better performance compared to well-known algorithms. The CAHBPSO method surpasses the BOA, PSO and semidefinite programming (SDP) algorithms for a broad spectrum of noise, according to simulation findings, and achieves the Cramer–Rao lower bound (CRLB).

## 1. Introduction

Determining the location of a passive target based on time difference of arrival (TDOA) measurements from multiple transmitters and a single receiver is a key element in many technologies, such as radar or sonar, telecommunications, mobile communications [1,2], etc. In general, two groups of localization approaches, active and passive, may be distinguished. The active localization approach takes into account the scenario when in the localization the target is actively involved. However, in the second group, the target does not participate in the localization process and merely serves to reflect the transmitter’s signals [3]. The global positioning system (GPS) has been widely used to determine the position of an object in outdoor environments [4]. However, this localization system cannot provide satisfactory performance in indoor, underwater acoustics, and urban environments, where the satellite signals are unavailable [5,6]. Therefore, passive target localization has become widely used in various applications, as an effective alternative to the GPS and other conventional localization systems.

Hence, the localization of a passive target is considered in this paper, where the noisy TDOA measurements are employed. The range measurements are calculated from the difference in the time it takes for a signal coming from a transmitter via the target to a receiver and the time required for a signal coming directly from the transmitter to a receiver. Therefore, the unknown position of a target becomes difficult to estimate since the TDOA measurements are nonlinear. The noisy TDOA measurements in the line-of-sight (LOS) environment can be described as the normal-distributed Gaussian random variable. Since the probability distribution of measurement error is known, the maximum likelihood (ML) estimator can be employed to estimate a passive target’s unknown position [2]. The ML estimator’s objective function is very nonlinear and non-convex, making it challenging to find the global optimal solution. Therefore, several efficient optimization algorithms are proposed in the literature to solve this type of optimization problem. Among these algorithms, the semidefinite programming (SDP) method became widely applied as it can efficiently transform the considered problem to convex optimization problem [2]. The SDP method’s primary benefit is that it does not demand a starting solution to solve the considered optimization problem. Furthermore, it can be solved employing MATLAB toolbox CVX with the SeDuMi solver. Still, the SDP method shows some drawbacks, which reflects on the accuracy of the obtained solution, primarily when the large measurement noise is present [7]. This shows that the solving of the non-convex ML estimation problem is an important and significant challenge.

In this context, evolutionary algorithms (EAs) are proposed with the aim to successfully achieve the global optimum to the challenging ML estimation problem, and get beyond the aforementioned drawbacks [8,9]. Generally speaking, there are two steps to the optimization process for EAs: global exploration and local exploitation [10]. Here, the first stage is concerned with identifying the area of the global optimum, and the second stage is concerned with increasing the convergence speed and the solution accuracy. Therefore, to efficiently find the global optimal solution, it is important to maintain a balance between exploration and exploitation during the optimization process of EAs.

Numerous EAs are proposed to solve different optimization problems, such as genetic algorithm (GA) [11], particle swarm optimization (PSO) [12], butterfly optimization algorithm (BOA) [13], differential evolution (DE) [14], cuckoo search algorithm (CS) [15], artificial bee colony (ABC) [16] and firefly algorithm (FA) [17], etc. During recent years, BOA and PSO algorithms are successfully applied to find the global optimal solution of different complex optimization problems, due to their advantages, such as easy implementation, fast convergence, and robustness [18].

The BOA is a novel optimization algorithm, developed by Arora and Singh [13], which is inspired by the foraging and mating behavior of butterflies. During the food searching process, the butterflies emit a fragrance, and the intensity of this fragrance is proportional to the objective function value of the butterfly. Other butterflies in the swarm can sense the fragrance intensity, in order to determine the potential direction of a food source or mating partner. In general, during the optimization process, the BOA algorithm goes through two phases: the global search phase and the local search phase. In the first phase, the BOA algorithm goes through the search space and finds regions where potential global solutions exist. Conversely, in the local search phase, the BOA algorithm performs a fine search in the neighborhood of the current optimal solution. Generally, the optimization performance of the BOA algorithm is influenced by the suitable choice of two control parameters: the sensory fragrance and power exponent [13]. The BOA algorithm is successfully applied to solve a wide range of optimization problems in science and engineering, where it has demonstrated better results compared to other EAs [18,19]. It has been shown that the BOA algorithm has a strong exploitation ability; however, it suffers from drawbacks, such as premature convergence to local optima and weak global search ability [20]. Therefore, to overcome these drawbacks, a number of improved versions of the BOA algorithm have been proposed in the literature [21,22].

The PSO algorithm is another nature-inspired EA, proposed by Kennedy and Eberhart [23], which is based on the social behavior of flocks of birds searching for food. The search process of the PSO algorithm is based on the position and velocity vectors. In this regard, each particle changes its position with respect to its personal previous best position and the best position of the whole swarm. Due to the easy implementation and effectiveness, the PSO algorithm is successfully applied to solve a wide range of complex optimization problems [24,25]. However, in solving complex optimization problems the PSO algorithm shows some disadvantages such as slow convergence rate and the problem of premature convergence [26]. It is shown in the literature that the performance of the PSO algorithm strongly depends on the appropriate choice of three control parameters: the inertia weight (*w*), the cognitive acceleration coefficient ($c_{1}$), and the social acceleration coefficient ($c_{2}$) [27]. In order to overcome these drawbacks and improve the performance, a number of adaptive and self-adaptive versions of the PSO algorithm are developed [28,29]. Furthermore, in recent years, the integration of chaos theory with the PSO algorithm has proven to be an effective way to improve the optimization performance and avoid the problem of premature convergence to local optima.

During the recent years, with the development of nonlinear dynamics, chaos theory is widely employed to improve different aspects of optimization algorithms [30]. Chaos is the bounded dynamic behavior of nonlinear systems characterized by infinite unstable periodic motions [31]. In this way, chaos maps are employed as an evolution function representing the chaos behavior, which produces a bounded sequence of random numbers depending on the choice of the initial conditions. A large number of chaos maps are found in the literature, such as the sinusoidal map, Chebyshev map, tent map, sine map, logistic map, etc. [32]. Due to the pseudo-randomness, ergodicity, and irregularity of chaos maps, the integration of chaos maps into EAs has been proven to be an effective way to improve the optimization performance and solution quality [21,29].

Another way to overcome the drawbacks and improve the optimization performance is the hybridization of different EAs. In this regard, the BOA algorithm is successfully hybridized with the PSO algorithm, in HPSOBOA, with the aim to improve convergence during the evolution process [33]. Moreover, the combination of search ability of the FA and PSO algorithms is proposed in the HFPSO algorithm, to improve the convergence speed and solution accuracy [34]. Additionally, different hybridizations of the BOA algorithm with other EAs, such as ABC and BOA (BOA/ABC) [16], BOA with DE (HBODEA) [35] and BOA with PSO (HPSO) [36] can be found in the literature, which are successfully applied to solve different complex optimization problems. Therefore, the hybridization of the EAs is proven to enhance the speed of reaching optimal solution, so as to avoid the problem of prematurely converging to a solution and provide more precise solutions.

This paper proposes a hybridization of the BOA and PSO algorithms, called CAHBPSO, to efficiently tackle the presented problem of localization of a passive target. In the proposed hybrid algorithm, the global and local search phases of the BOA algorithm are incorporated into the velocity update equation of the PSO algorithm. In addition, instead of fixed-switch probability, an adaptive technique is proposed to dynamically switch between exploration and exploitation. To further improve the performance, an adaptive strategy is employed to update the sensory fragrance of the BOA algorithm. Moreover, the logistic chaos map is incorporated into the inertia weight parameter of the PSO algorithm, to maintain a trade-off between local and global abilities.

The localization performance is measured against the Cramer-Rao lower bound (CRLB), which offers a lower constraint on the unbiased estimator variance [37]. Therefore, the root mean square error (RMSE) performance of the new CAHBPSO method and the current SDP, BOA, and PSO algorithms are compared with the derived CRLB. The following is a summary of this paper’s significant contributions:

- The problem of localization of a passive target is formulated using noisy TDOA measurements obtained from a set of transmitters and a single receiver, for the case of LOS conditions. Due to the highly nonlinear and non-convex nature of the ML estimation problem that has been formulated for the consideration localization problem, sophisticated optimization algorithms are proposed to address this complex optimization problem.
- By converting the considered multimodal optimization problem to a problem with distinct single extremum, the SDP method, as a convex method, is employed to effectively address the ML estimation problem.
- The enhanced CAHBPSO algorithm—a hybridization of the BOA and the PSO algorithms—is proposed, to precisely estimate the position of the passive target. To improve convergence and maintain population diversity the global and local search phases of the BOA algorithm are incorporated into the velocity update equation of the PSO algorithm. In addition, instead of fixed-switch probability, an adaptive parameter is employed to effectively maintain a trade-off between global and local search abilities throughout the iteration process. Furthermore, the sensory fragrance of the BOA algorithm is adaptively updated and logistic chaos map is incorporated into the expression for the inertia weight parameter of the PSO algorithm.
- The Wilcoxon signed-rank test and Friedman test are employed for statistical performance comparison between CAHBPSO algorithm with several widely applied EAs on a set of CEC2014 problems. Analyzing the optimization performance, according to the statistical analysis’s findings the modifications and hybridization proposed in this paper successfully enhance the CAHBPSO algorithm.
- The results of the numerical simulation demonstrate that the proposed CAHBPSO method outperforms SDP, BOA, and PSO algorithms in terms of localization performance and CRLB accuracy. Furthermore, according to the simulation findings, the CAHBPSO method performs the best when there is a high level of measurement noise and it is not sensitive to changes in network layout. In terms of computational complexity, the simulation results showed that the proposed algorithm provides a proper balance between localization accuracy and complexity compared to other considered algorithms.

The remainder of this paper is structured as follows. An overview of the existing literature is given in Section 2. The TDOA-based problem of passive target localization is presented in Section 3. In Section 4, the function for the problem of passive target localization based on ML estimation is derived. The formulation of the SDP method is described in Section 5. In Section 6, the conventional BOA algorithm with the improved version is described. The conventional PSO algorithm with the proposed modification of the inertia weight improved with the logistic chaos map is presented in Section 7. The proposed CAHBPSO algorithm is presented in Section 8. Section 9 gives the formulation of the CRLB for the considered passive target localization problem. Section 10 provides the numerical simulation results. The conclusions and directions for future work are drawn in Section 11. Finally, the derivation of the CRLB is given in Appendix A.

## 2. Background and Related Work

Accurate estimation of the location of a passive target in the presence of additive measurement noise has become an important and challenging issue [38]. The estimated target position’s accuracy is mostly determined by two factors: measurement precision and the geometric layout of receivers, transmitters, and target [39]. The two types of localization algorithms that can be roughly categorized are range-based and range-free algorithms. Range-based localization algorithms estimate a target’s position effectively by using distance or angle information between the target and receivers. These methods are based on data collected using a variety of ranging approaches, including time of arrival (TOA) [38], TDOA [17], received signal strength (RSS) [40], angle of arrival (AOA) [41] and their combinations [42]. Because of its capacity to produce high localization accuracy, the TDOA is one of the most extensively utilized techniques [43]. In contrast to range-based localization algorithms, range-free localization techniques estimate the unknown node position using connectivity and topological information [44]. Distance vector hop (DV-Hop) [45], centroid or weighted centroid technique [46], and approximation point-in-triangulation test (APIT) [47] are the most extensively used range-free algorithms. Because these methods do not require a sophisticated hardware structure to determine range measurements, they are both inexpensive and simple to build. However, when compared to range-based algorithms, range-free algorithms typically have inferior localization accuracy [48].

The nonlinear least squares (NLS) estimator [49], which is obtained by minimizing the sum of the squared measurement errors, is a widely used estimation method for obtaining the unknown position of a target. Generally, the solution in closed form for NLS estimator is not obtainable, making it a challenge to obtain the solution of NLS nonlinear and non-convex objective function. To obtain the closed-form solution of different localization problems the linear least squares (LLS) and weighted least squares (WLS) methods are often applied [50]. However, these methods do not provide the required localization accuracy, and thus can be applied to provide an initial solution to the iterative optimization methods.

In this way, a number of local search optimization algorithms are widely applied to solve different localization problems [51]. We differentiate between the two groups of optimization algorithms, whether or not they require knowledge of the gradient of objective function, both of which perform local searches around the given starting solution [52]. Hence, numerous local search algorithms, such as Gauss–Newton, Nelder–Mead, and Conjugate gradient method are employed to estimate the unknown target position by solving the NLS estimation problem. However, finding the global optimum using local search optimization algorithms highly depends on the provided initial solution [52]. This leads to the conclusion that the convergence of local search algorithms while handling multimodal optimization problems is typically not expected without suitable initialization.

Another widely used estimation method is the ML estimator, which is commonly applied when the measurement error distribution is previously known [53]. However, because of the nonlinearity and non-convexity of the ML estimator’s objective function, typical local search techniques cannot be used to address this sort of complicated optimization problem. In this regard, to formulate a convex problem, second-order cone programming (SOCP) and SDP methods are widely applied to transform ML estimation problem, overcoming the non-convexity of the ML objective function [54]. In comparison to the SOCP method, the simulation results of the comparative study of both algorithms reveal that the SDP method gives superior precision of the target location [55]. The SDP and SOCP methods, on the other hand, cannot the achieve desired precision of the estimated solution, especially in the cases when severe measurement noise is present. As a result, there is a lot of interest in improving and developing efficient EAs that can be used to identify the global best solution to the nonlinear and non-convex ML estimation problem.

Various EAs, such as CS, PSO, BOA, and FA, etc., are applied to solve different localization problems by estimating the unknown location of a target [9,18,56]. As a result, finding an effective optimization algorithm for a specific localization problem is critical in order to reduce the localization error in all situations. A significant increase on the positioning accuracy is achieved by using PSO and TDOA together [57]. Results are compared with well-known WLS and LLS techniques and the results reveal that the PSO method outperforms the well-known methods. Furthermore, the PSO algorithm based on chaos theory is proposed for the hybrid TDOA/AOA location estimation problem, where the objective function is formulated using the ML estimator [9]. The comparative analysis shows that the improved PSO algorithm has enhanced global search ability compared to the conventional optimization algorithms. For the localization of wireless sensor nodes in “concave areas”, researchers suggested a two-stage PSO technique. The approach can achieve excellent localization accuracy in wireless sensor networks while using little energy and processing resources [58]. In [18], the BOA algorithm is employed to estimate the location of nodes by minimizing the considered objective function. The results of the localization performance of BOA demonstrate more consistent and accurate location of nodes compared to widely applied methos, such as PSO and FA.

In addition, the hybridization of EAs is proven to be as an effective way to enhance the quality of the solutions and improve the optimization efficiency. In this way, using TDOA measurements a hybrid firefly algorithm (hybrid-FA), which combines the WLS and FA algorithms, is proposed in [17] for target coordinates estimation. According to the simulation findings, the hybrid-FA method outperforms other well-known localization algorithms in terms of performance and localization accuracy. In addition, various hybrid variants of EAs, such as PSO and variable neighborhood search (HPSOVNS) [59], DE and FA (HFDLA) [60] are successfully applied to enhance positioning accuracy.

Based on the preceding, an improved CAHBPSO algorithm is proposed here to increase the accuracy of the estimated target position, especially when measurement noise increases.

## 3. Localization Problem

**Figure 1:**
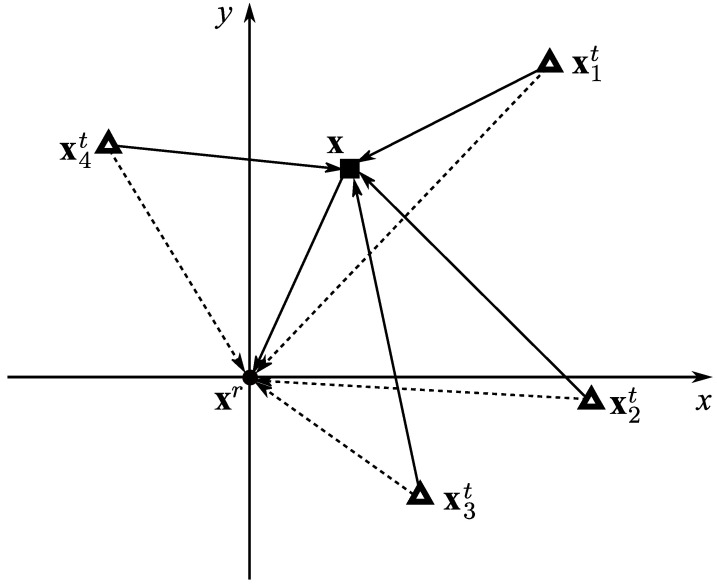
Passive target localization using noisy TDOA measurements.

This section looks at the problem of determining the unknown location of a passive target in a LOS environment using noisy TDOA measurements. As shown in Figure 1, the investigated localization system consists of one receiver at the origin of the coordinate system $x_{r}={\left[\begin{array}{cc} 0 & 0 \end{array}\right]}^{T},$ *N* transmitters with predefined known coordinates $x_{i}^{t}={\left[\begin{array}{cc} x_{i}^{t} & y_{i}^{t} \end{array}\right]}^{T}\in R^{2},$$\;\forall i\in \left\{1,2,\cdots ,N\right\}$, and the unknown position of the passive target at $x={\left[\begin{array}{cc} x & y \end{array}\right]}^{T}\in R^{2}$.

In the passive target localization problem, the transmitters emit a signal, and the target reflects the signal from each of the transmitters in all directions. The TDOA measurements are then obtained by the receiver capturing the reflected signal as well as the direct signal from each of the transmitters. Furthermore, the receiver and transmitters are assumed to be perfectly synced, and it is assumed that transmitter emitted signals are reflected from the passive target across all possible directions while not engaging directly with the receiver and transmitters [37]. When modelling the measurement error it is valid to assume that its probability distribution is Gaussian, which holds in LOS conditions [2]. Therefore, the noisy TDOA measurements are

(1) $$t_{i}=\frac{1}{c}\left({∥x_{i}^{t}-x∥}_{2}+{∥x_{r}-x∥}_{2}-{∥x_{i}^{t}-x_{r}∥}_{2}\right)+{\bar{n}}_{i},\forall i\in \left\{1,2,\cdots ,N\right\},$$

where ${∥\;\cdot \;∥}_{2}$ is the Euclidean distance in two dimensions, *c* denotes a constant which is equal to the known speed of light, and ${\bar{n}}_{i}$ represents measurement noise whose underlying probability distribution, according to the assumption, is Gaussian. The range measurements ${\left\{r_{i}\right\}}_{i=1}^{N},$ can be derived by multiplying Equation (1) with constant *c*, as follows

(2) $$\begin{array}{cc} r_{i} & =c\cdot t_{i}\\ & =\left({∥x_{i}^{t}-x∥}_{2}+{∥x_{r}-x∥}_{2}-{∥x_{i}^{t}-x_{r}∥}_{2}\right)+n_{i},\forall i\in \left\{1,2,\ldots ,N\right\}, \end{array}$$

where $n_{i}=c{\bar{n}}_{i}$ follows the zero-mean Gaussian distribution. Then, introducing a new variable ${\tilde{r}}_{i}=r_{i}+{∥x_{i}^{t}-x_{r}∥}_{2}$, and substituting it into the range measurements in Equation  (2), the following expression can be obtained

(3) $${\tilde{r}}_{i}={∥x_{i}^{t}-x∥}_{2}+{∥x_{r}-x∥}_{2}+n_{i},\forall i\in \left\{1,2,\ldots ,N\right\}.$$

Then, the vector form of Equation (3) can be expressed as

(4) $$\tilde{r}=d\left(x\right)+n,$$

where

(5) $$d\left(x\right)=\left[\begin{array}{c} {∥x_{1}^{t}-x∥}_{2}+{∥x_{r}-x∥}_{2}\\ \vdots \\ {∥x_{N}^{t}-x∥}_{2}+{∥x_{r}-x∥}_{2} \end{array}\right],$$

and $n={\left[n_{1},n_{2},\ldots ,n_{N}\right]}^{T}$ is the vector of zero-mean Gaussian noise. It is assumed that all elements of n are independent and identically distributed. In this regard, the covariance matrix can be obtained as $C=E\left[nn^{T}\right]=\sigma^{2}I_{N}$ [37], where $E\left[\cdot \right]$ denotes the expectation operator and $I_{N}$ is $\left(N\times N\right)$ identity matrix. Then, the vector $d\left(x\right)$ can be rewritten as

(6) $$d\left(x\right)=Hg\left(x\right),$$

where

(7) $$g\left(x\right)={\left[\begin{array}{ccccc} ∥x_{r}-x∥ & ∥x_{1}^{t}-x∥ & & \ldots & ∥x_{N}^{t}-x∥ \end{array}\right]}^{T},$$

(8) $$H=\left[1_{N}I_{N}\right]=\left[\begin{array}{ccccc} 1 & 1 & \cdots & 0 & 0\\ 1 & 0 & \cdots & 0 & 0\\ \vdots & \vdots & \ddots & \vdots & \vdots \\ 1 & 0 & \cdots & 0 & 1 \end{array}\right],$$

in which $1_{N}$ denotes the unit column vector of length *N*. Then, Equation (4) can be reformulated as

(9) $$\tilde{r}=Hg\left(x\right)+n.$$

As a result, the goal of this paper is to solve the nonlinear and non-convex ML estimation problem and to effectively estimate the unknown position of the passive target x based on noisy TDOA data.

## 4. Maximum Likelihood Estimator

The Maximum likelihood estimator can be successfully employed to determine the unknown coordinates of the passive target by determining the extremum of the likelihood function. Under the assumption that TDOA measurements are independent and identically distributed Gaussian zero-mean random variables, the likelihood function $L\left(\tilde{r}\left|x\right.\right)$ of the obtained TDOA measurements can be expressed as

(10) $$\begin{array}{c} \begin{array}{cc} \; & L\left(\tilde{r}\left|x\right.\right)=f\left(\tilde{r}\left|x\right.\right)=\\ \; & =\frac{1}{{\left(2\pi \right)}^{N/2}\det{\left(C\right)}^{1/2}}\exp\left(-\frac{1}{2}{\left(\tilde{r}-Hg\left(x\right)\right)}^{T}C^{-1}\left(\tilde{r}-Hg\left(x\right)\right)\right), \end{array} \end{array}$$

where $f\left(\tilde{r}\left|x\right.\right)$ is the probability density function of the measurements. Then, taking the logarithm of the likelihood function yields

(11) $$\ln L\left(\tilde{r}\left|x\right.\right)=k-\frac{1}{2\sigma^{2}}\left({\left(\tilde{r}-Hg\left(x\right)\right)}^{T}\left(\tilde{r}-Hg\left(x\right)\right)\right),$$

where $k=\ln\left(\frac{1}{{\left(2\pi \right)}^{N/2}\det{\left(C\right)}^{1/2}}\right)$ can be neglected as it does not depend on x. In this regard, the estimated position of the passive target $\hat{x}$ is obtained as the solution of the following non-convex optimization problem

(12) $$\hat{x}=\underset{x\in R^{2}}{\mathrm{argmin}}\;\left(J_{ML}\left(x\right)\right),$$

where the ML objective function corresponding to this may be written as

(13) $$J_{ML}\left(x\right)={\left(\tilde{r}-Hg\left(x\right)\right)}^{T}\left(\tilde{r}-Hg\left(x\right)\right).$$

**Figure 2:**
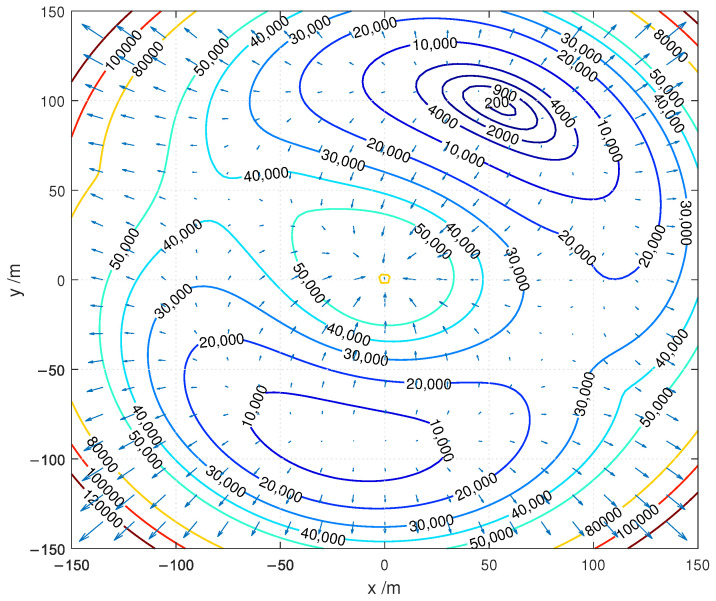
The contour plot of the considered ML objective function $J_{ML}\left(x\right)$.

Figure 2 shows the corresponding contour plot of the ML objective function $J_{ML}\left(x\right)$.

According to the plot of the the objective function $J_{ML}\left(x\right)$ in Figure 2 it is concluded that the $J_{ML}\left(x\right)$ is nonlinear function which has multiple local optima. Furthermore, it is noticed that the position of the global minimum of this function correlates to the coordinates of the unknown position of the target. As a result, advanced optimization methods, discussed in reminder of the paper, are required to obtain the global optimal solution.

## 5. Semidefinite Programming Method

This section presents the SDP approach to deal with non-convexity of the ML estimation problem by transforming it into a convex optimization problem in order to solve the passive target localization problem [2]. As described, the considered localization problem leads to the non-convex and multimodal ML objective function. In order to try to solve this problem, the SDP method is developed for the considered passive target TDOA-based localization problem, which converts the objective function $J_{ML}\left(x\right)$ to a convex function. The ML estimation problem Equation (12) with regard to x can be expressed as follows:

(14) $$\hat{x}=\underset{x\in R^{2}}{\arg\min}\;{\left(\tilde{r}-Hg\left(x\right)\right)}^{T}\left(\tilde{r}-Hg\left(x\right)\right).$$

The objective function of the considered ML optimization problem can be transformed into a set of linear equations, by squaring both sides of Equation (2) and introducing an additional variable between the receiver and the target $R^{r}=∥x_{r}-x∥$, as follows

(15) $$\begin{array}{c} -2{\left(x_{r}-x_{i}^{t}\right)}^{T}x-2{\tilde{r}}_{i}R^{r}+{\tilde{r}}_{i}^{2}+{∥x_{r}∥}^{2}-{∥x_{i}^{t}∥}^{2}\\ =2\left({\tilde{r}}_{i}-R^{r}\right)n_{i},\forall i\in \left\{1,2,\ldots ,N\right\}, \end{array}$$

where the second-order term of the noise $n_{i}^{2}$ is neglected. Introducing the variable θ, as

(16) $$\theta ={\left[\begin{array}{cc} x & R^{r} \end{array}\right]}^{T},$$

the Equation (15) can be expressed in the linear-matrix from

(17) b−Aθ=m,

where

(18) $$A=2\left[\begin{array}{ccc} x_{r}-x_{1}^{t} & y_{r}-y_{1}^{r} & {\tilde{r}}_{1}\\ x_{r}-x_{2}^{t} & y_{r}-y_{2}^{r} & {\tilde{r}}_{2}\\ \vdots & \vdots & \vdots \\ x_{r}-x_{N}^{t} & y_{r}-y_{N}^{r} & {\tilde{r}}_{N} \end{array}\right],$$

(19) $$b=\left[\begin{array}{c} {\tilde{r}}_{1}^{2}+x_{r}^{2}+y_{r}^{2}-{\left(x_{1}^{t}\right)}^{2}-{\left(y_{1}^{t}\right)}^{2}\\ {\tilde{r}}_{2}^{2}+x_{r}^{2}+y_{r}^{2}-{\left(x_{2}^{t}\right)}^{2}-{\left(y_{2}^{t}\right)}^{2}\\ \vdots \\ {\tilde{r}}_{N}^{2}+x_{r}^{2}+y_{r}^{2}-{\left(x_{N}^{t}\right)}^{2}-{\left(y_{N}^{t}\right)}^{2} \end{array}\right],$$

and

(20) $$m=2\left[\begin{array}{c} \left({\tilde{r}}_{1}-R^{r}\right)n_{1}\\ \left({\tilde{r}}_{2}-R^{r}\right)n_{2}\\ \vdots \\ \left({\tilde{r}}_{N}-R^{r}\right)n_{N} \end{array}\right].$$

Then, based on Equation (16) through Equation (20), the following WLS optimization problem can be formulated as

(21) $$\min_{\;\;\;\theta \;\;\;}\;J\left(\;\;\;\theta \;\;\;\right)=\min_{\;\;\;\theta \;\;\;}\;{\left(b-A\theta \right)}^{T}W\left(b-A\theta \right)$$

where $W\in R^{N\times N}$ is a symmetric weighting matrix. Under the sufficiently small measurement noise, the symmetric weighting matrix can be approximated as

(22) $$W={\left[E\left\{mm^{T}\right\}\right]}^{-1}={\left(D^{T}CD\right)}^{-1},$$

where

(23) $$D=\mathrm{diag}\left\{2\left({\tilde{r}}_{1}-R^{r}\right),2\left({\tilde{r}}_{2}-R^{r}\right),\ldots ,2\left({\tilde{r}}_{N}-R^{r}\right)\right\},$$

It should be noted that since the measurement noise m from Equation (20) is Gaussian distributed and due to the linear relationship in Equation (17), the objective function of the ML estimator, given in Equation (13), is equivalent to that of the WLS estimator in Equation (21) [61].

Then, by introducing the range between $x_{r}$ and x, denoted by $R^{r}={∥x_{r}-x∥}_{2}$ as the equality constraint, the ML estimation problem in Equation (14) is expressed as

(24) $$\begin{array}{c} \begin{array}{cc} \; & \min_{x}\;f_{ob}=\min_{\;\;\;\theta \;\;\;}\;{\left(b-A\theta \right)}^{T}W\left(b-A\theta \right)\\ \; & s.t.\;R^{r}={∥x_{r}-x∥}_{2}. \end{array} \end{array}$$

After corresponding algebraic manipulation, the objective function of the optimization problem $f_{ob}$ in Equation (24) becomes

(25) $$f_{ob}=\sum_{i=1}^{N}\frac{{\left(b_{i}-a_{i}\theta \right)}^{2}}{{\left({\tilde{r}}_{i}-R^{r}\right)}^{2}}={\sum_{i=1}^{N}\left[\frac{b_{i}}{{\tilde{r}}_{i}-R^{r}}-\frac{{\tilde{a}}_{i}\theta }{{\tilde{r}}_{i}-R^{r}}-\frac{\nu_{i}R^{r}}{{\tilde{r}}_{i}-R^{r}}\right]}^{2},$$

where $b_{i}$ denotes the *i*th element of vector b, $a_{i}$ is the *i*th row of matrix A. Here, $a_{i}=\left[\begin{array}{cc} {\tilde{a}}_{i} & \nu_{i} \end{array}\right]$ denotes a block matrix, where the submatrices are ${\tilde{a}}_{i}=\left[\begin{array}{ccc} a_{i}\left(1\right) & \ldots & a_{i}\left(n\right) \end{array}\right]$ and $\nu_{i}=a_{i}\left(n+1\right)$.

To transform the objective function, the matrix property $x^{T}\mathrm{Ax}=\mathrm{Tr}\left(\;xx^{T}A\;\right)$ is employed. After introduction of the matrix notation $z=x^{T}x$, the Equation (25) can be rewritten as

(26) $$\begin{array}{c} \begin{array}{cc} \; & {\left[\begin{array}{c} x\\ 1 \end{array}\right]}^{T}\left[\begin{array}{cc} \sum_{i=1}^{N}\frac{{\tilde{a}}_{i}\tilde{a}{{}_{i}^{T}}_{i}}{{\left({\tilde{r}}_{i}-R^{r}\right)}^{2}} & \sum_{i=1}^{N}\frac{{\tilde{a}}_{i}\left(\nu_{i}R^{r}-b_{i}\right)}{{\left({\tilde{r}}_{i}-R^{r}\right)}^{2}}\\ \sum_{i=1}^{N}\frac{{\tilde{a}}_{i}^{T}\left(\nu_{i}R^{r}-b_{i}\right)}{{\left({\tilde{r}}_{i}-R^{r}\right)}^{2}} & \sum_{i=1}^{N}\frac{{\left(\nu_{i}R^{r}-b_{i}\right)}^{2}}{{\left({\tilde{r}}_{i}-R^{r}\right)}^{2}} \end{array}\right]\left[\begin{array}{c} x\\ 1 \end{array}\right]=\\ \; & =\mathrm{Tr}\left(P\left[\begin{array}{cc} x^{T} & 1 \end{array}\right]\left[\begin{array}{c} x\\ 1 \end{array}\right]\right)=\mathrm{Tr}\left(P\;\;\left[\begin{array}{cc} z & x\\ x^{T} & 1 \end{array}\right]\right), \end{array} \end{array}$$

in which

(27) $$P=\left[\begin{array}{cc} \sum_{i=1}^{N}\frac{{\tilde{a}}_{i}\tilde{a}{{}_{i}^{T}}_{i}}{{\left({\tilde{r}}_{i}-R^{r}\right)}^{2}} & \sum_{i=1}^{N}\frac{{\tilde{a}}_{i}\left(\nu_{i}R^{r}-b_{i}\right)}{{\left({\tilde{r}}_{i}-R^{r}\right)}^{2}}\\ \sum_{i=1}^{N}\frac{{\tilde{a}}_{i}^{T}\left(\nu_{i}R^{r}-b_{i}\right)}{{\left({\tilde{r}}_{i}-R^{r}\right)}^{2}} & \sum_{i=1}^{N}\frac{{\left(\nu_{i}R^{r}-b_{i}\right)}^{2}}{{\left({\tilde{r}}_{i}-R^{r}\right)}^{2}} \end{array}\right],$$

and $\mathrm{Tr}\left(\cdot \right)$ denotes the trace of a square matrix.

Then, based on Equations (26) and (27) the optimization problem in Equation (24) can be equivalently written as

(28) $$\begin{array}{c} \begin{array}{cc} \; & \min_{x,z}\;\mathrm{Tr}\left(P\;\;\left[\begin{array}{cc} z & x\\ x^{T} & 1 \end{array}\right]\right)\\ \; & s.t.\;R^{r}={∥x_{r}-x∥}_{2}\\ \; & \;\;z=x^{T}x. \end{array} \end{array}$$

The constraint in Equation (28) can be reformulated using the notation $z=x^{T}x$, as follows

(29) $$\begin{array}{c} \begin{array}{cc} \; & {R^{r}}^{2}={∥x_{r}-x∥}_{2}^{2}=x_{r}^{T}x_{r}-x_{r}^{T}x-x^{T}x_{r}+x^{T}x\\ \; & \Leftrightarrow {R^{r}}^{2}=\mathrm{Tr}\left\{\left[\begin{array}{cc} I_{n} & -x_{r}\\ -{x_{r}}^{T} & {x_{r}}^{T}x_{r} \end{array}\right]\left[\begin{array}{cc} x^{T} & 1 \end{array}\right]\left[\begin{array}{c} x\\ 1 \end{array}\right]\right\}\\ \; & =\mathrm{Tr}\left\{\left[\begin{array}{cc} I_{n} & -x_{r}\\ -{x_{r}}^{T} & {x_{r}}^{T}x_{r} \end{array}\right]\left[\begin{array}{cc} z & x\\ x^{T} & 1 \end{array}\right]\right\}. \end{array} \end{array}$$

Finding the global optimal solution of the optimization problem in Equation (28) is difficult due to the non-convex equality constraint $z=x^{T}x$. Therefore, the equality constraint $z=x^{T}x$ is relaxed to a convex constraint as  

(30) $$z-x^{T}x≽0,$$

Then, after applying the Schur complement [62], the constraint can be equivalently rewritten as a linear matrix inequality, as follows

(31) $$\left[\begin{array}{cc} z & x\\ x^{T} & 1 \end{array}\right]≽0.$$

Hence, the obtained matrix in Equation (31) is symmetric and positive semidefinite matrix. Finally, the optimization problem in Equation (28) can be phrased as follows, based on the above relaxation.

(32) $$\begin{array}{c} \begin{array}{cc} \; & \min_{x,z,R^{r}}\;\mathrm{Tr}\left(P\;\;\left[\begin{array}{cc} z & x\\ x^{T} & 1 \end{array}\right]\right)\\ \; & s.t.\;{R^{r}}^{2}=\mathrm{Tr}\left\{\left[\begin{array}{cc} I_{n} & -x_{r}\\ -{x_{r}}^{T} & {x_{r}}^{T}x_{r} \end{array}\right]\left[\begin{array}{cc} z & x\\ x^{T} & 1 \end{array}\right]\right\}\\ \; & \;\;\left[\begin{array}{cc} z & x\\ x^{T} & 1 \end{array}\right]≽0. \end{array} \end{array}$$

It should be noted, that for each fixed $R^{r}$, the obtained problem in Equation (32) becomes convex optimization problem. However, this optimization problem becomes non-convex, when the Equation (32) is solved with respect to variables $\left\{x,z,R^{r}\right\}$, e.g., when the value of $R^{r}$ is not previously known. Therefore, it is important to find the optimal value of $R^{r}$, for which the problem in Equation (32) becomes convex. Thus, in this paper, the golden section optimization algorithm (GSA) [52] is employed in order to determine the optimal value of $R^{r}$. In this regard, two points within the interval $\left[R_{l}^{r},R_{u}^{r}\right]$ can be calculated using the equations

(33) $$\begin{array}{c} \begin{array}{cc} \; & R_{1}^{r}=R_{l}^{r}+\left(1-\varphi \right)\left(R_{u}^{r}-R_{l}^{r}\right),\\ \; & R_{2}^{r}=R_{l}^{r}+\varphi \left(R_{u}^{r}-R_{l}^{r}\right) \end{array} \end{array}$$

where $\varphi =\left(-1+\sqrt{5}\right)/2\;$ represents the golden ratio. Afterwards, for each point $R_{j}^{r}$, j=1,2 obtained in Equation (33), the solutions $x_{j}$ and $z_{j}$ of the optimization problem in Equation (32) are found, and the objective function can be evaluated at these points. If $f_{ob}\left(x_{1},z_{1},R_{1}^{r}\right)<f_{ob}\left(x_{2},z_{2},R_{2}^{r}\right)$, then the optimal point belongs to the interval $\left[R_{l}^{r},R_{2}^{r}\right]$, otherwise if $f_{ob}\left(x_{1},z_{1},R_{1}^{r}\right)>f_{ob}\left(x_{2},z_{2},R_{2}^{r}\right)$ the solution belongs to $\left[R_{1}^{r},R_{u}^{r}\right]$. Alternatively, if $f_{ob}\left(x_{1},z_{1},R_{1}^{r}\right)=f_{ob}\left(x_{2},z_{2},R_{2}^{r}\right)$ the boundaries are reduced to $R_{l}^{r}=R_{1}^{r}$ and $R_{u}^{r}=R_{2}^{r}$, and the optimization process is repeated. The values of $R_{1}^{r}$ and $R_{2}^{r}$ are calculated iteratively until the difference $\left|R_{1}^{r}-R_{2}^{r}\right|\leq \varepsilon$ is less than a predefined positive number ε.

Therefore, the procedure for determining the optimal value of $R^{r},$ for which the considered SDP optimization problem becomes convex problem can be stated as follows:

Step 1: Initialize the $R_{l}^{r}$ and $R_{u}^{r}$.
Step 2: Calculate the points $R_{1}^{r}$ and $R_{2}^{r}$ according to Equation (33).
Step 3: Solve Equation (32) with $R^{r}=R_{1}^{r}$ and $R^{r}=R_{2}^{r}$.
Step 4: If $f_{ob}\left(x_{1},z_{1},R_{1}^{r}\right)<f_{ob}\left(x_{2},z_{2},R_{2}^{r}\right)$, then the interval becomes $\left[R_{l}^{r},R_{2}^{r}\right]$, otherwise interval is $\left[R_{1}^{r},R_{u}^{r}\right]$. If $f_{ob}\left(x_{1},z_{1},R_{1}^{r}\right)=f_{ob}\left(x_{2},z_{2},R_{2}^{r}\right)$ boundaries become $R_{l}^{r}=R_{1}^{r}$ and $R_{u}^{r}=R_{2}^{r}$ and go to Step 2.
Step 5: Repeat Steps 2–4 until $\left|R_{1}^{r}-R_{2}^{r}\right|\leq \varepsilon$ is satisfied.

## 6. Butterfly Optimization Algorithm and the Proposed Improved Version

### 6.1. Conventional BOA Algorithm

The butterfly optimization algorithm is a novel nature-inspired metaheuristic algorithm, where the search process is inspired by the food foraging behavior and the process of mating between butterflies [13]. The BOA is based on three assumptions:

1. All butterflies are said to release some fragrance in order to attract one another.
2. Each butterfly either moves randomly or towards the butterfly with the strongest fragrance (i.e., the best butterfly in the current generation)
3. The stimulus intensity of a butterfly is proportional to the objective function value.

In general, the optimization process of the BOA algorithm can be divided into three stages: initialization, iteration, and the final optimization stage. In the first stage, the values of control parameters of the algorithm are defined, and the set of initial solutions is randomly generated within the upper and lower bounds. In the iteration stage, the search for the global optimal solution is performed. Firstly, the algorithm calculates the objective function value of each butterfly, and then butterflies generate the fragrance at their positions. The intensity of the fragrance sensed by the butterfly, $\varphi_{i}$, can be described as a function of the physical intensity of stimulus, as follows

(34) $$\varphi_{i}=cI^{a},$$

where *c* denotes the sensory fragrance, *a* represents the power exponent, and *I* is the stimulus intensity, which is proportional to the objective function value. Here, coefficients *c* and *a* are assigned in the range $\left[0,1\right]$. In the case a=1, there is no fragrance absorption, while when a=0, other butterflies cannot detect the fragrance produced by any butterfly. Stimulus intensity *I* for *i*th butterfly can be determined as

(35) $$I=f\left(x_{i}^{\left(G\right)}\right),$$

where $x_{i}^{\left(G\right)}$ denotes the position of the *i*th butterfly in the *G*th generation, and *f* is the objective function of the considered optimization problem.

During the search for the global optimum, the BOA algorithm goes through two key phases, the global search phase and the local search phase. In the first phase, the butterfly is updating its position according to the global best solution

(36) $$x_{i}^{\left(G+1\right)}=x_{i}^{\left(G\right)}+\left(r^{2}\times g^{*}-x_{i}^{\left(G\right)}\right)\times \varphi_{i},$$

where $g^{*}$ represents the best solution found in the current iteration (i.e., butterfly with the strongest fragrance) and *r* represents uniform random number in the range $r\in \left[0,1\right]$. On the other hand, the local search phase of the BOA algorithm can be written as

(37) $$x_{i}^{\left(G+1\right)}=x_{i}^{\left(G\right)}+\left(r^{2}\times x_{j}^{\left(G\right)}-x_{k}^{\left(G\right)}\right)\times \varphi_{i},$$

where $x_{j}^{\left(G\right)}$ and $x_{k}^{\left(G\right)}$ are the *j*th and *k*th butterfly positions within the search space, respectively.

The switch probability *p* is used to transition between the global and local search phases of the algorithm, as shown below

(38) $$\left\{\begin{array}{cc} x_{i}^{\left(G+1\right)}=x_{i}^{\left(G\right)}+\left(r^{2}\times g^{*}-x_{i}^{\left(G\right)}\right)\times \varphi_{i}, & \mathrm{if}\;r_{p}<p\\ x_{i}^{\left(G+1\right)}=x_{i}^{\left(G\right)}+\left(r^{2}\times x_{j}^{\left(G\right)}-x_{k}^{\left(G\right)}\right)\times \varphi_{i}, & \mathrm{otherwise} \end{array}\right.$$

where $r_{p}$ denotes uniformly generated random number in the range $\left[0,1\right]$. Therefore, the global search phase of the BOA algorithm, defined in Equation (36), is applied if $r_{p}<p$. Otherwise, the local search phase, given in Equation (37), is employed to search for the optimal solution. The above-mentioned process is repeated until the stopping criteria is satisfied and the optimal solution is obtained, in the final optimization stage of the algorithm.

### 6.2. Improved BOA Algorithm

The conventional BOA algorithm provides an excellent local search ability; however, it suffers from premature convergence to local optima due to its poor exploitation ability [20]. Therefore, there is a need to modify the trade-off between the global and local search of the BOA in order to solve multimodal and complex optimization problems.

Analyzing Equation (34), it is evident that sensory fragrance *c* is one of the most important parameters in BOA, which guides the movement of butterflies during the search process by enabling each butterfly to sense the fragrances emitted by other butterflies. Therefore, using a constant value for parameter *c* is not suitable for complex optimization problems. Using a small value of *c*, during the entire search process, can result in premature convergence. On the other hand, in the early stage of the search process, a large constant value of sensory fragrance *c* leads to a high probability of missing the area of global optimal solution, which reduces the optimization performance. In this regard, it is critical to adjust the value of the sensory fragrance *c* adaptively during the search process in order to enhance the balance between exploration and exploitation ability.

**Figure 3:**
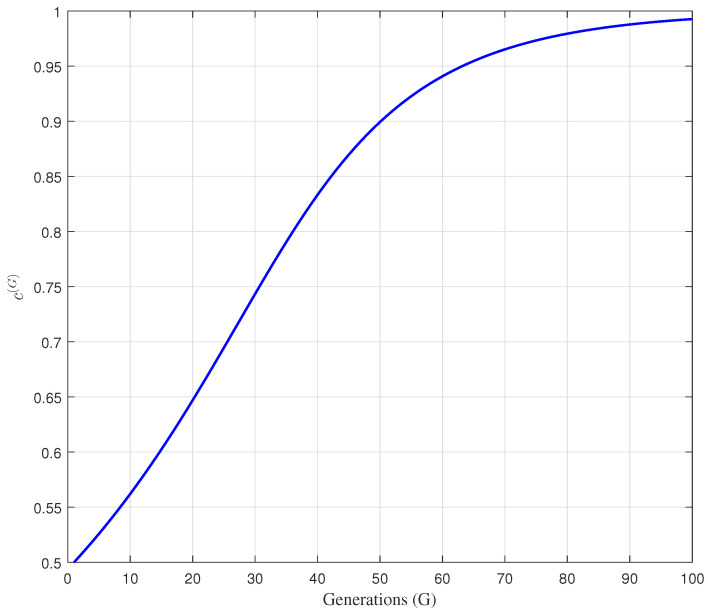
The changes of the adaptive sensory fragrance $c^{\left(G\right)}$ during the optimization process.

Therefore, to provide an appropriate balance between global and local search abilities, in this paper, an adaptive sensory fragrance $c^{\left(G+1\right)}$ has been introduced, which can be described as follows

(39) $$c^{\left(G+1\right)}=\frac{1}{1+\exp\left(-\frac{c^{\left(G\right)}G}{0.2G_{\max}}\right)},$$

where $G_{\max}$ denotes the maximum number of generations. In this respect, the changes of proposed adaptive sensory fragrance $c^{\left(G+1\right)}$, defined in Equation (39), versus the number of generations is illustrated in Figure 3.

According to Figure 3, it can be observed that the adaptive sensory fragrance $c^{\left(G\right)}$ has smaller value at the beginning of the search process, and achieves a larger value with the increase of generations. Therefore, in the early stage of the search process, the smaller value of $c^{\left(G\right)}$ can enhance the global exploration ability and prevent premature convergence. On the other hand, in the later stage of search process, a larger value of $c^{\left(G\right)}$ can improve exploitation ability and convergence speed of the algorithm.

## 7. Particle Swarm Optimization and the Proposed Improved Version

### 7.1. Conventional PSO Algorithm

In the PSO algorithm [23], the search process is performed based on velocity and position vectors. Each particle in a swarm of $N_{P}$ particles at *G*th generation, has a position vector $x_{i}^{\left(G\right)}={\left[x_{i,1}^{\left(G\right)},x_{i,2}^{\left(G\right)},\ldots ,x_{i,n}^{\left(G\right)}\right]}^{T}$ and velocity vector $v_{i}^{\left(G\right)}={\left[v_{i,1}^{\left(G\right)},v_{i,2}^{\left(G\right)},\ldots ,v_{i,n}^{\left(G\right)}\right]}^{T},$$\forall i\in \left\{1,2,\ldots ,N_{P}\right\}$, in the *n*-dimensional space. In the initial generation, the position and velocity vectors are randomly generated within the upper and lower bounds. During the optimization process, each particle moves through the search space with velocity $v_{i}^{\left(G\right)}$, which depends on the personal previous best position $p_{i}^{\left(G\right)}={\left[p_{i,1}^{\left(G\right)},p_{i,2}^{\left(G\right)},\ldots ,p_{i,n}^{\left(G\right)}\right]}^{T}$ and best position discovered by the whole population $g_{}^{\left(G\right)}={\left[g_{1}^{\left(G\right)},g_{2}^{\left(G\right)},\ldots ,g_{n}^{\left(G\right)}\right]}^{T}$. Therefore, the position and velocity vectors of each particle at $\left(G+1\right)$th generation are updated as follows

(40) $$v_{i}^{\left(G+1\right)}=v_{i}^{\left(G\right)}+c_{1}r_{1}\left(p_{i}^{\left(G\right)}-x_{i}^{\left(G\right)}\right)+c_{2}r_{2}\left(g_{}^{\left(G\right)}-x_{i}^{\left(G\right)}\right),$$

(41) $$x_{i}^{\left(G+1\right)}=x_{i}^{\left(G\right)}+v_{i}^{\left(G+1\right)},$$

where $c_{1}$ and $c_{2}$ are the cognitive and social acceleration coefficients, respectively, which are commonly set to 2 [26]. Here, $r_{1}$ and $r_{2}$ are two distinct random numbers uniformly distributed in the range $\left[0,1\right]$. In order to ensure the balance between the exploration and exploitation abilities during the optimization process, the inertia weight $\omega^{\left(G\right)}$ is introduced into the PSO algorithm. Therefore, the velocity vector $v_{i}^{\left(G+1\right)}$ of each particle is updated using the linear-decreasing inertia [63], as follows

(42) $$v_{i}^{\left(G+1\right)}=\omega^{\left(G\right)}v_{i}^{\left(G\right)}+c_{1}r_{1}\left(p_{i}^{\left(G\right)}-x_{i}^{\left(G\right)}\right)+c_{2}r_{2}\left(g_{}^{\left(G\right)}-x_{i}^{\left(G\right)}\right),$$

(43) $$\omega^{\left(G\right)}=\omega_{\max}-\frac{G}{G_{\max}}\left(\omega_{\max}-\omega_{\min}\right),$$

where $\omega_{\max}$ and $\omega_{\min}$ denote the maximum and minimum values of the inertia weight, respectively. In the literature, the maximum and minimum value of inertia weight is commonly set to $\omega_{\max}=0.9$ and $\omega_{\min}=0.4$ [63].

### 7.2. Chaos Enhanced PSO Algorithm

The conventional PSO algorithm exhibits the issue of premature convergence to local optima, which may affect its optimization performance in solving complex optimization problems [26]. In this regard, the introduction of chaos theory into the PSO algorithm can improve the optimization performance, by modifying the PSO algorithm to escape more easily from local optima [30,64,65]. Therefore, in this paper, the logistic chaos map is implemented to dynamically adjust the value of inertia weight, in order to maintain the appropriate balance between global exploration and local exploitation abilities during the optimization process.

#### Chaotic Dynamic Inertia Weight

It is well known in the literature that the inertia weight has an important role in maintaining the balance between exploitation and exploration abilities during the optimization process [27]. Analyzing Equation (43), it is evident that a large value of inertia weight, in the early stage of the search process, can improve the global search abilities of the PSO algorithm and prevent the problem of premature convergence. On the other hand, a smaller value of inertia weight, in the later stage of search process, can improve the local search ability of the PSO algorithm. A number of different inertia weight strategies are proposed in the literature, among which the linear-decreasing inertia weight given in Equation (43) is widely employed. According to the analysis [27,66], choosing the appropriate inertia weight strategy for the current optimization problem depends on the properties of the objective function. In order to obtain an appropriate balance between exploration and exploitation skills for solving complex optimization problems, additional modifications to the inertia weight are necessary.

In recent years, the introduction of chaos theory is emerging as a powerful approach for improving the optimization performance of different metaheuristic algorithms [64,65]. Chaos is a bounded dynamic behavior that can be observed in certain nonlinear dynamic systems. In this regard, the chaotic behavior can be represented with chaos maps, which produce a bounded sequence of random numbers depending on initial condition. In this way, the well-known logistic chaotic map [65], which has the properties of ergodicity, non-repetition and irregularity, is employed in this paper, to adjust the value of inertia weight. The expression for logistic map is given as

(44) $$\begin{array}{c} x_{c}^{\left(G+1\right)}=a_{\omega }x_{c}^{\left(G\right)}\left(1-x_{c}^{\left(G\right)}\right),\;G=1,2,\ldots ,G_{\max}. \end{array}$$

where $a_{\omega }$ denotes the control parameter. It should be noted that for certain initial conditions, e.g., $x_{c}^{\left(0\right)}\notin \left\{0,0.25,0.5,0.75,1\right\}$ and $a_{\omega }=4$, the logistic chaos map exhibits chaotic behavior.

**Figure 4:**
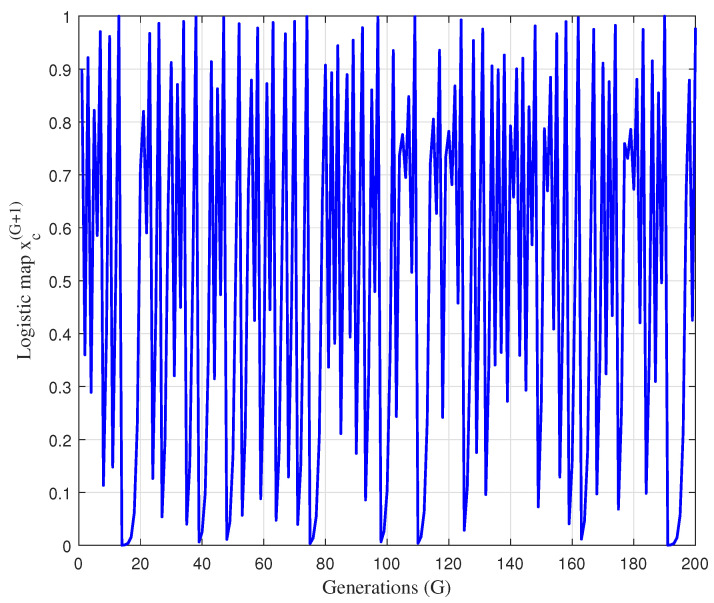
The the unbounded chaotic behavior produced by logistic chaotic map for the $x_{c}^{\left(0\right)}=0.9$ and $a_{\omega }=4$.

In Figure 4, the unbounded chaotic behavior produced by logistic chaotic map, given in Equation (44) is presented as a function of generation number *G*, for the $x_{c}^{\left(0\right)}=0.9$ and $a_{\omega }=4$.

Furthermore, to better control the inertia weight parameter, the logistic chaos map is upper-bounded by introducing the term $e^{-G/G_{\max}\;}$. Therefore, the chaotic dynamic inertia weight $\omega_{c}^{\left(G+1\right)}$ can be calculated as follows

(45) $$\begin{array}{c} \omega_{c}^{\left(G\right)}=e^{-\frac{G}{G_{\max}}}\cdot x_{c}^{\left(G\right)} \end{array}$$

**Figure 5:**
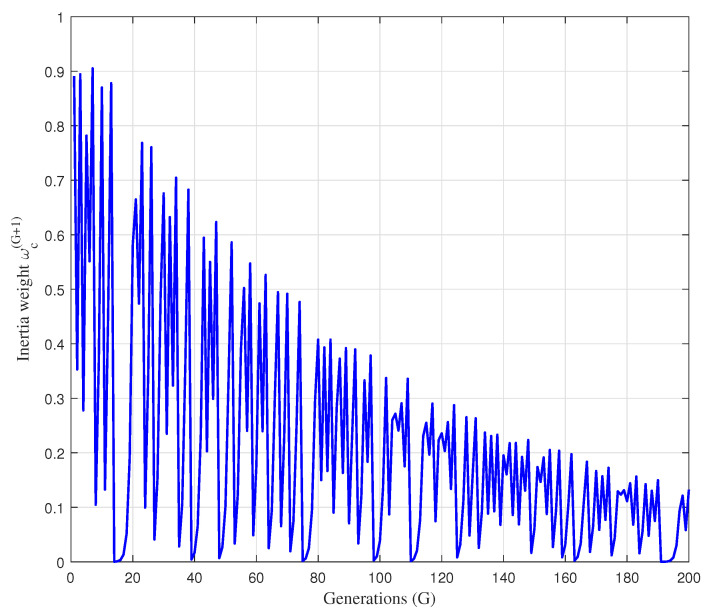
The changes of the chaotic dynamic inertia weight $\omega_{c}^{\left(G+1\right)}$ during the search process.

In this respect, Figure 5 illustrates the changes of the proposed chaotic dynamic inertia weight $\omega_{c}^{\left(G+1\right)}$, defined in Equation (45), with the increase of generations.

As can be seen from Figure 5, the chaotic dynamic inertia weight $\omega_{c}^{\left(G+1\right)}$, defined in Equation (45), is upper-bounded by the exponential factor $e^{-G/G_{\max}\;}$, and the value of $\omega_{c}^{\left(G+1\right)}$ gradually decreases with the increase of generations. In the early stage of the search process, $\omega_{c}^{\left(G+1\right)}$ has a larger value, which is suitable for enhancing the global search ability and finding the region of the global optimal solution. In the later stage of search process, a smaller value of $\omega_{c}^{\left(G+1\right)}$ can enhance the exploitation ability and improve the convergence towards the global optimum. This shows that the proposed chaotic dynamic inertia weight $\omega_{c}^{\left(G+1\right)}$ provides an effective balance between global exploration and local exploitation abilities, and thus improves the optimization performance of the PSO algorithm.

## 8. Chaos Enhanced Adaptive Hybrid Butterfly Particle Swarm Optimization Algorithm

To efficiently solve the considered complex passive target localization problem, in this section, CAHBPSO algorithm is introduced, as a hybridization of the BOA with PSO algorithm. In order to overcome the problem of premature convergence and enhance the exploration and exploitation abilities of PSO algorithm, in this paper, the global search and local search phases of conventional BOA algorithm are incorporated into the velocity update equation of the PSO algorithm. Instead of fixed switch probability, we propose an adaptive technique to dynamically adjust between global exploration and local exploitation abilities during the optimization process. In addition, the proposed adaptive strategy for updating the value of the sensory fragrance of the BOA algorithm is introduced into the CAHBPSO algorithm. Furthermore, the chaotic dynamic inertia weight, proposed in this paper, is incorporated into the hybrid algorithm with the aim to improve convergence and efficiently maintain the population diversity. Therefore, to improve the optimization performance and achieve a more efficient algorithm for complex optimization problems, the following two modifications are proposed.

Firstly, the exploration of the PSO algorithm is enhanced by replacing the term $c_{2}r_{2}\left(g^{\left(G\right)}-x_{i}^{\left(G\right)}\right)$ in Equation (42) with $\left(r^{2}\times g^{*}-x_{i}^{\left(G\right)}\right)\times \varphi_{i}$ from the global search phase of the BOA algorithm, given in Equation (36). Furthermore, $v_{i}^{\left(G\right)}$ in Equation (42) is substituted with $x_{i}^{\left(G\right)}$ from Equation (36), and linear inertia weight $\omega^{\left(G\right)}$ is replaced with chaotic dynamic inertia weight $\omega_{c}^{\left(G+1\right)}$. The equation for updating the *i*th butterfly’s location can thus be expressed as

(46) $$x_{i}^{\left(G+1\right)}=\omega_{c}^{\left(G+1\right)}x_{i}^{\left(G\right)}+c_{1}^{}r_{1}\left(p_{i}^{\left(G\right)}-x_{i}^{\left(G\right)}\right)+\left(r^{2}\times g^{*}-x_{i}^{\left(G\right)}\right)\times \varphi_{i}.$$

Next, further modification is done with the aim to enhance the local search ability PSO algorithm. In this regard, we exchange the term $c_{1}r_{1}\left(p_{i}^{\left(G\right)}-x_{i}^{\left(G\right)}\right)$ in Equation (42) with the term $\left(r^{2}\times x_{j}^{\left(G\right)}-x_{k}^{\left(G\right)}\right)\times \varphi_{i}$ from the local search phase of the BOA algorithm, given in Equation (37). Furthermore, $v_{i}^{\left(G\right)}$ in Equation (42) is substituted with $x_{i}^{\left(G\right)}$ from Equation (37) and the proposed chaotic dynamic inertia weight $\omega_{c}^{\left(G+1\right)}$ is introduced. Therefore, the new butterfly position is determined by

(47) $$x_{i}^{\left(G+1\right)}=\omega_{c}^{\left(G+1\right)}x_{i}^{\left(G\right)}+\left(r^{2}\times x_{j}^{\left(G\right)}-x_{k}^{\left(G\right)}\right)\times \varphi_{i}+c_{2}r_{2}\left(g^{\left(G\right)}-x_{i}^{\left(G\right)}\right).$$

In this paper, instead of using a fixed switch probability, an adaptive mechanism is proposed to dynamically adjust between global exploration and local exploitation during the optimization process. In this regard, the adaptive switch probability $p^{\left(G+1\right)}$ can be described as follows

(48) $$p^{\left(G+1\right)}=\left|\frac{f_{mean}^{\left(G\right)}-f_{best}^{\left(G\right)}}{f_{worst}^{\left(G\right)}-f_{best}^{\left(G\right)}}\right|,$$

where $f_{mean}^{\left(G\right)}$, $f_{best}^{\left(G\right)}$ and $f_{worst}^{\left(G\right)}$ denote the mean, best, and worst values butterflies achieved in the previous generation in terms of objective function, respectively.

The trade of between global and local search abilities during the optimization process can be managed by changing the parameter $p^{\left(G+1\right)}$, according to Equation (48). In the following analysis we can consider two two extreme cases. Firstly, the parameter $p^{\left(G+1\right)}$ is near to 1, indicating that the algorithm’s global exploration capabilities has to be improved since the diversity in the population is low. As a result, Equations (36) and (46) will be picked at random with a probability of 0.5, with the goal of improving global exploration and locating the global optimal solution region. In the second case, parameter $p^{\left(G+1\right)}$ is close to 0, indicating that all the solutions are near the global optimal solution, which shows that local exploitation must be improved. Therefore, Equations (37) and (47) will be randomly selected, with the probability of 0.5, to enhance exploitation ability and improve the convergence speed.

In this regard, the position of the ith butterfly can be updated based on the value of parameter $p^{\left(G+1\right)}$ according to the pseudocode shown in Algorithm 1.

**Algorithm 1** Position update of the *i*th butterfly of the proposed hybrid CAHBPSO algorithm.    **if** 
$p^{\left(G+1\right)}>0.5$ 
**then**        **if** rand>0.5 **then**            $x_{i}^{\left(G+1\right)}=x_{i}^{\left(G\right)}+\left(r^{2}\times g^{*}-x_{i}^{\left(G\right)}\right)\times \varphi_{i}$        **else**            $x_{i}^{\left(G+1\right)}=\omega_{c}^{\left(G+1\right)}x_{i}^{\left(G\right)}+c_{1}^{\left(G\right)}r_{1}\left(p_{i}^{\left(G\right)}-x_{i}^{\left(G\right)}\right)+\left(r^{2}\times g^{*}-x_{i}^{\left(G\right)}\right)\times \varphi_{i}$        **end if**    **else if**  $p^{\left(G+1\right)}\leq 0.5$ 
**then**        **if** rand>0.5 **then**            $x_{i}^{\left(G+1\right)}=x_{i}^{\left(G\right)}+\left(r^{2}\times x_{j}^{\left(G\right)}-x_{k}^{\left(G\right)}\right)\times \varphi_{i}$        **else**            $x_{i}^{\left(G+1\right)}=\omega_{c}^{\left(G+1\right)}x_{i}^{\left(G\right)}+\left(r^{2}\times x_{j}^{\left(G\right)}-x_{k}^{\left(G\right)}\right)\times \varphi_{i}+c_{2}^{\left(G\right)}r_{2}\left(g^{\left(G\right)}-x_{i}^{\left(G\right)}\right)$        **end if**    **end if**

The proposed modifications, introduced in the CAHBPSO algorithm, provide an effective balance between exploration and exploitation abilities during the optimization process. Furthermore, these modifications are effective in overcoming the problem of premature convergence. In this way, the pseudocode of the proposed CAHBPSO algorithm is presented in Algorithm 2, for the considered passive target localization problem.

**Algorithm 2** Pseudo-code of the proposed CAHBPSO algorithm.    Generate initial population    Determine stimulus intensity $I_{i}=f\left(x_{i}^{\left(0\right)}\right)$    Set the value of parameters $c_{1}$, $c_{2}$, *a*, $G_{max}$, $a_{\omega }$, $\omega^{\left(0\right)}$    Initialize values of $p_{i}^{\left(0\right)}$ and $g^{\left(0\right)}$    **while** stopping criteria not met **do**        Calculate adaptive sensory modality function $c^{\left(G\right)}$ according to Equation (39)        **for** each butterfly *i* in the population **do**            Calculate fragrance $\varphi_{i}=cI^{a}$        **end for**        Find the best butterfly        Calculate chaotic inertia weight according to Equation (45)        **for** each butterfly *i* in the population **do**            **if** $p^{\left(G+1\right)}>0.5$ **then**               **if** rand>0.5 **then**                   $x_{i}^{\left(G+1\right)}=x_{i}^{\left(G\right)}+\left(r^{2}\times g^{*}-x_{i}^{\left(G\right)}\right)\times \varphi_{i}$               **else**                   $x_{i}^{\left(G+1\right)}=\omega_{c}^{\left(G+1\right)}x_{i}^{\left(G\right)}+c_{1}^{\left(G\right)}r_{1}\left(p_{i}^{\left(G\right)}-x_{i}^{\left(G\right)}\right)+\left(r^{2}\times g^{*}-x_{i}^{\left(G\right)}\right)\times \varphi_{i}$               **end if**            **else if** $p^{\left(G+1\right)}\leq 0.5$ **then**               **if** rand>0.5 **then**                   $x_{i}^{\left(G+1\right)}=x_{i}^{\left(G\right)}+\left(r^{2}\times x_{j}^{\left(G\right)}-x_{k}^{\left(G\right)}\right)\times \varphi_{i}$               **else**                   $x_{i}^{\left(G+1\right)}=\omega_{c}^{\left(G+1\right)}x_{i}^{\left(G\right)}+\left(r^{2}\times x_{j}^{\left(G\right)}-x_{k}^{\left(G\right)}\right)\times \varphi_{i}+c_{2}^{\left(G\right)}r_{2}\left(g^{\left(G\right)}-x_{i}^{\left(G\right)}\right)$               **end if**            **end if**        **end for**        Find the global best solution $g^{\left(G\right)}$        Determine the personal best solution according to:        $p_{i}^{\left(G+1\right)}=\left\{\begin{array}{c} p_{i}^{\left(G\right)},\;\;\;\;\mathrm{if}\;p_{i}^{\left(G\right)}≺x_{i}^{\left(G\right)}\\ x_{i}^{\left(G\right)},\;\mathrm{otherwise} \end{array}\right.$    **end while**

## 9. Cramer–Rao Lower Bound

The CRLB for the passive target localization problem provides a lower bound on the covariance matrix of any unbiased estimator [37]. Therefore, in this paper, the CRLB is used as a benchmark to evaluate the performance of the considered estimators. The derivation of the CRLB can be obtained from the inverse of the Fisher information matrix (FIM) $F\left(x\right)$, which can be defined as

(49) $$\begin{array}{c} \begin{array}{cc} \; & F\left(x\right)=E\left[\left(\frac{\partial \ln\left(f\left(\tilde{r}\left|x\right.\right)\right)}{\partial x}\right){\left(\frac{\partial \ln\left(f\left(\tilde{r}\left|x\right.\right)\right)}{\partial x}\right)}^{T}\right]\\ \; & =-E\left[\frac{\partial^{2}\ln\left(f\left(\tilde{r}\left|x\right.\right)\right)}{\partial x\partial x^{T}}\right]. \end{array} \end{array}$$

Thus, the components of the FIM are given as follows

(50) $$F\left(x\right)=\left[\begin{array}{cc} F_{11} & F_{12}\\ F_{21} & F_{22} \end{array}\right],$$

where the corresponding elements can be obtained as

(51) $$F_{11}=\frac{1}{\sigma^{2}}\sum_{i=1}^{N}{\left(\frac{x-x_{r}}{∥x-x_{r}∥}+\frac{x-x_{i}^{t}}{{∥x-x_{i}^{t}∥}_{2}}\right)}^{2},$$

(52) $$\begin{array}{c} \begin{array}{cc} F_{12}=F_{21} & =\frac{1}{\sigma^{2}}\sum_{i=1}^{N}\left(\frac{x-x_{r}}{∥x-x_{r}∥}+\frac{x-x_{i}^{t}}{{∥x-x_{i}^{t}∥}_{2}}\right)\\ \; & \times \left(\frac{y-y_{r}}{∥x-x_{r}∥}+\frac{y-y_{i}^{t}}{{∥x-x_{i}^{t}∥}_{2}}\right), \end{array} \end{array}$$

(53) $$F_{22}=\frac{1}{\sigma^{2}}\sum_{i=1}^{N}{\left(\frac{y-y_{r}}{∥x-x_{r}∥}+\frac{y-y_{i}^{t}}{{∥x-x_{i}^{t}∥}_{2}}\right)}^{2}.$$

The derivations of Equations (51)–(53) are given in Appendix A. Then, the relationship between the variance and CRLB can be defined as

(54) $$E\left[\left(\hat{x}-x\right)\;{\left(\hat{x}-x\right)}^{T}\right]\geq \mathrm{Tr}\left\{F{\left(x\right)}^{-1}\right\}=\mathrm{CRLB}\left(x\right),$$

where $\hat{x}$ denotes the estimated value of x.

## 10. Experimental Study

This section present the experimental results conducted in order to evaluate the localization accuracy of the proposed CAHBPSO algorithm and compare the optimization performance with the well-known algorithms in the literature on a set of CEC2014 benchmark problems using the statistical analysis. Therefore, the obtained results are outlined in the following two subsections.

### 10.1. Statistical Evaluation of CAHBPSO Method against the CEC2014 Benchmark

This section outlines the results of the statistical comparison of the optimization performance between the proposed CAHBPSO algorithm and widely applied algorithms in the literature, including PSO [23], BOA [13], SHADE [67] and HPSOBOA [33] on a set of CEC2014 benchmark problems. The CEC2014 benchmark problems consist of 30 single-objective real-parameter numerical optimization problems, where D=10,30,50, and 100 are the considered dimensions of the search space, which are defined in [68]. The considered CEC2014 benchmark problems can be classified into four groups:

- $f_{1}-f_{3}$ unimodal optimization problems;
- $f_{4}-f_{16}$ simple multimodal objective functions;
- $f_{17}-f_{22}$ hybrid objective functions, in which variables are subdivided and various basic functions are applied to each subset;
- $f_{23}-f_{30}$ composition functions, which provide continuity around the optimal solution and merge the properties of sub-functions.

The metric of the obtained solution error $f\left(\hat{x}\right)$–$f\left(x^{*}\right)$ was established to make a statistical comparison of the optimization performance of CAHBPSO and other widely applied algorithms, where $\hat{x}$ represents the global optimum of each algorithm produced in a single run and $x^{*}$ denotes the solution to the CEC2014 benchmark problem, which is previously known. For each of the test functions, each algorithm was run 51 times with the termination threshold set at 10,000*D* and the swarm size was $N_{P}=100$ particles. The search space for each objective function is defined as [−100100]D. As a consequence, the obtained experimental findings are examined and compared using two nonparametric statistical hypothesis tests, such as the Wilcoxon signed-rank test and the Friedman test, to perform statistical assessment of optimization performance.

To perform pair-wise comparison of optimization performance between the proposed CAHBPSO and other well-known algorithms, the Wilcoxon signed-rank test is applied. In this regard, statistical comparison findings can reveal if the first algorithm outperforms the second method statistically. The significance level for this statistical test was set at 0.05. In the findings, $R^{+}$ means the total of rankings in which the first algorithm exceeded the second, whereas $R^{-}$ denotes the sum of ranks in which the second algorithm outperformed the first algorithm. The Wilcoxon signed-rank test’s null hypothesis asserts that “there is no difference between the mean findings of the two samples” [69]. The alternative hypothesis, on the other hand, asserts that “there is a difference in the mean results of the two samples”. Therefore, using the *p* value and comparing it with the significance level α, the null hypothesis can be rejected when p≤α. In this regard, the obtained results are denoted with signs +, ≈, − according to the result of the statistical test. Here, plus sign (+) denotes that the first algorithm had significantly better optimization performance than second one, minus sign (−) indicates that the first algorithm performed significantly worse than the first one, while the sign (≈) denotes that there is no significant difference in optimization performance between two considered algorithms.

The Friedman test is used in this study to determine the substantial difference between the optimization performances of the studied methods. In order to determine the rankings of all examined algorithms across each CEC2014 objective function over each of the search space dimensions *D*, the Friedman test is utilized. As a result, the algorithm with the lowest rank performs the best in terms of optimization, while the method with the highest rank performs the worst. The Friedman test’s null hypothesis is that “there is no difference among the performance of all algorithms”, whereas the alternative hypothesis is that “there is a difference among the performance of all algorithms” [69]. When the *p* value is less than or equal to α=0.05, the null hypothesis can be rejected.

The numerical simulations results of the proposed CAHBPSO and other considered algorithms, computed over 51 independent trials on a set of CEC2014 benchmark problems are presented in Table 1. The results are presented in terms of mean (Mean) and standard deviation (STD) of the best obtained objective function value. Furthermore, the sign is added to indicate if the examined method outperforms (+), performs similarly (≈), or worse (−) than the suggested CAHBPSO method.

Based on the findings in Table 1, it is clear that the proposed CAHBPSO algorithm outperforms other studied algorithms in terms of “Mean” and “STD” values on the vast majority of benchmark functions. However, compared to the PSO algorithm, the proposed algorithm achieved a worse performance on simple multimodal functions $f_{4},f_{13}$ on dimension D=30, $f_{12},f_{16}$ on dimensions D=50,100, and $f_{11}$ on D=100. Furthermore, a worse performance is observed on hybrid function $f_{22}$ on dimension D=100 and on composite functions $f_{24},f_{27}$ on dimensions D=30,50,100, as well as functions $f_{24},f_{25},f_{26}$ on dimensions D=100, function $f_{26}$ over dimension D=10, and $f_{28}$ on dimensions D=30. Compared to the BOA algorithm, the proposed CAHBPSO algorithm showed a worse performance on composite functions $f_{23}$ and $f_{27}$ on all dimensions, $f_{24},f_{25},f_{29},f_{30}$ on dimensions D=30,50,100, and function $f_{26}$ on dimensions D=50,100. When compared to the SHADE and FA algorithms, it is clear that CAHBPSO outperforms them on all functions across all dimensions. Only on the hybrid and composite functions $f_{18}$, D=10 and $f_{26}$, D=50 did the proposed algorithm perform worse than the SHADE algorithm. As a result, when compared to the SHADE and FA algorithms, the suggested CAHBPSO algorithm delivers the best results. The proposed algorithm performed similarly to or slightly worse than the BOA and PSO algorithms on problems with greater dimensions, such as D=30,50 and 100 on composite functions. However, the suggested method surpassed the BOA and PSO algorithms on the majority of test functions.Compared to the HPSOBOA algorithm, it is observed that the proposed algorithm achieved worse performance over all considered dimensions on functions $f_{6}$, $f_{10}$, $f_{11}$, $f_{13}$, $f_{14}$, $f_{28}$ and $f_{30}$, while the CAHBPSO algorithm outperformed HPSOBOA method on functions $f_{15}$, $f_{16}$, $f_{17}$, $f_{18}$, $f_{19}$, $f_{20}$, $f_{21}$, $f_{22}$ and $f_{29}$ over all dimensions. On other functions the results are mixed, depending on the dimensionality of the problem. It can be observed that when dimension D=10 is concerned the CAHBPSO algorithm achieved the best result in the majority of the results compared to HPSOBOA. As a conclusion, the CAHPBSO method’s improvements and hybridization are confirmed, and the CAHBPSO algorithm effectively discovers potential solutions across all CEC2014 benchmark tests and dimensions.

The obtained numerical results are analyzed using Wilcoxon signed-rank test, in order to perform the pair-wise optimization performance comparison between the proposed CAHBPSO and other considered algorithms. Therefore, the results of statistical comparison using Wilcoxon’s signed-rank test on CEC2014 benchmark problems are presented in Table 2.

From the results in Table 2, it can be observed that the proposed CAHBPSO algorithm has significantly better performance than the SHADE, FA, BOA, and PSO algorithms on all considered dimensions. Compared to the BOA and PSO algorithms, on D=30,50, and 100 an increase is observed in cases where the proposed algorithm had worse performance. When comparing to the HPSOBOA algorithm, we observe that the proposed CAHBPSO algorithm achieved better performance for D=10, while for other dimensions the algorithms had similar performance. However, in all considered cases, the CAHBPSO algorithm achieved higher $R^{+}$ values than $R^{-}$ compared to considered algorithms.

Furthermore, the Friedman test is used to assess whether there is a significant statistical difference in the optimization performances of the algorithms considered. Table 3 displays the average ranks obtained by the Friedman test of examined algorithms on various CEC2014 problems over all dimensions. The algorithm’s top rank is bolded, while the second best is underlined.

From the statistical comparison results presented in Table 3, it can be observed that the proposed CAHBPSO algorithm achieves the best performance among the considered algorithms and has the lowest rank in all considered cases. The HPSOBOA algorithm had the second-best performance. It is observed that the obtained *p* values for the considered Friedman test are less than the significance level α=0.05 in all dimensions over all considered cases. As a result, the hypothesis suggests that there is a considerable discrepancy in the performance of the algorithms under consideration. Based on the statistical analysis findings, it is determined that the proposed hybridization of BOA and PSO algorithms increases optimization performance while overcoming the disadvantages of both techniques. Furthermore, the enhanced approach for updating butterfly positions, as well as the addition of a chaotic map to the inertia weight and adaptive sensory fragrance, considerably increase the CAHBPSO algorithm capabilities, demonstrating the efficacy of proposed improvements.

### 10.2. Localization Performance of the Proposed CAHBPSO Algorithm

In this section, simulations are performed to verify and compare the localization performance of the proposed CAHBPSO with the existing algorithms, such as SDP, PSO, BOA, and the well-known WLS method [50], for the considered passive target localization problem in LOS environment. Here, the derived CRLB is utilized as a performance benchmark in the following simulations to assess the localization capabilities in terms of RMSE metric.

The simulations are performed to evaluate the localization accuracy of all considered algorithms under the noisy TDOA measurements. Furthermore, the performance of different algorithms is assessed in relation to changes in transmitter, target, and receiver geometric configurations. The numerical simulations are carried out using a passive target localization system consisting of one receiver and four transmitters positioned at known locations, as well as a passive target located at various locations. Three simulation scenarios are considered in this regard, depending on the chosen coordinates where the passive target is placed: (i) the passive target is surrounded by the four transmitters; (ii) the target is outside the hull which are transmitters-forming; and (iii) the passive target is deployed randomly over an area $80\times 80\;m^{2}$ for each simulation run. The identical receiver and transmitter setup is used in each simulation scenario, with the receiver at $x_{r}={\left[\begin{array}{cc} 0 & 0 \end{array}\right]}^{T},$ and four transmitters creating a convex hull at $x_{1}^{t}={\left[\begin{array}{cc} 80 & 80 \end{array}\right]}^{T}$m, $x_{2}^{t}={\left[\begin{array}{cc} 80 & -80 \end{array}\right]}^{T}$m, $x_{3}^{t}={\left[\begin{array}{cc} -80 & 80 \end{array}\right]}^{T}m,$ and $x_{4}^{t}={\left[\begin{array}{cc} -80 & -80 \end{array}\right]}^{T}$m. Therefore, to evaluate the localization accuracy of the considered algorithms, the RMSE is employed, which is defined as

(55) $$\mathrm{RMSE}=\sqrt{\frac{1}{N_{m}}\sum_{n=1}^{N}{∥{\hat{x}}^{\left(n\right)}-x∥}_{2}^{2}},$$

where x denotes the is the actual target location, ${\hat{x}}^{\left(n\right)}$ represents the position of a target that is estimated using one of the considered algorithms and $N_{m}=1000$ is the number of Monte Carlo simulation repetitions for a given variance of measurement noise $\sigma_{}^{2}$.

**Figure 6:**
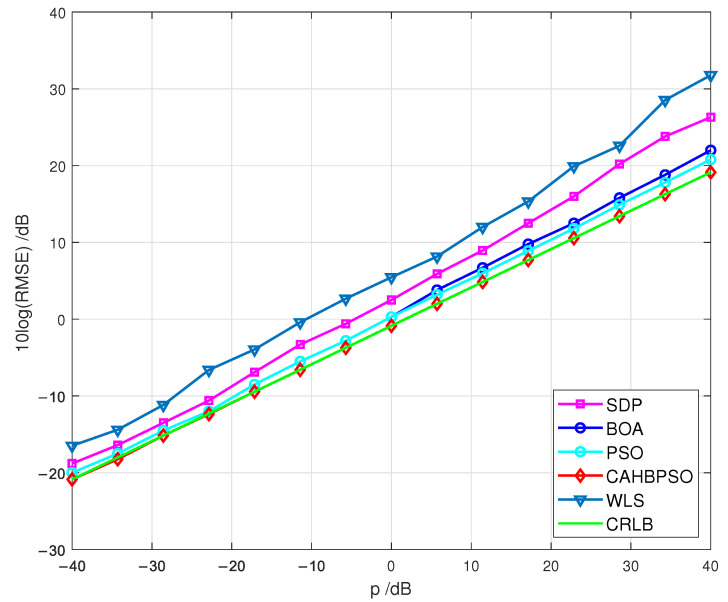
RMSEs in terms of *p* for a passive target at $x={\left[\begin{array}{cc} 20 & 30 \end{array}\right]}^{T}$.

Figure 6 shows the results of the first simulation scenario, in which the true position of the passive target is $x={\left[\begin{array}{cc} 20 & 30 \end{array}\right]}^{T}$m. The RMSE performances of the examined algorithms are displayed in relation to measurement noise $p=10\log\left(\sigma_{}^{2}\right)$ and compared to the CRLB.

The RMSEs obtained using the CAHBPSO method achieve the CRLB for the whole studied ranges of *p*, as shown in Figure 6. Furthermore, the RMSE performances of the WLS, SDP, BOA, and PSO algorithms are several dBs higher than the CRLB. However, the obtained numerical results reveal that the SDP approach deviates significantly from the CRLB for large values of *p* (esp. *p* > 20 dB) when compared to examined algorithms. It is observed that the RMSEs of the well-known WLS method show the worst performance over all considered methods.

**Figure 7:**
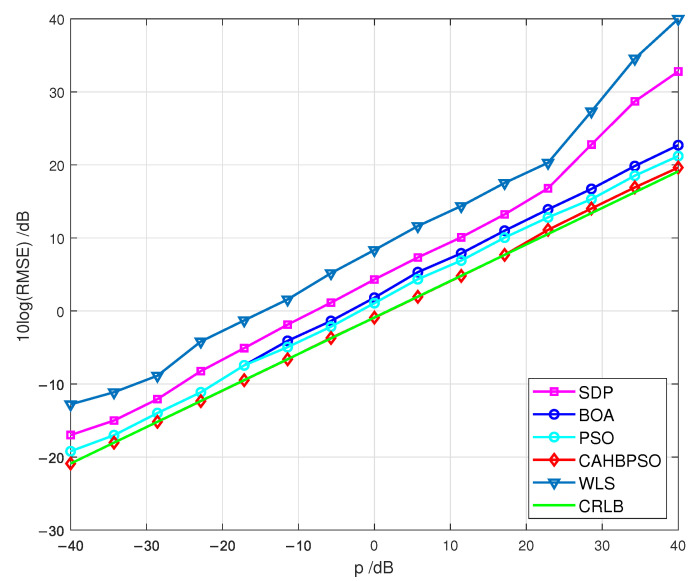
RMSEs in terms of *p* for a passive target at $x={\left[100\;80\right]}^{T}$.

The findings of the second simulation scenario, in which the true location of the target is placed at $x={\left[100\;80\right]}^{T}$m, are presented in Figure 7, where the RMSE performances of the studied algorithms are plotted and compared with the CRLB.

According to the results in Figure 7, the proposed CAHBPSO method achieves CRLB accuracy and outperforms the investigated algorithms with the increase of *p*. In comparison to the CAHBPSO method, the BOA and PSO algorithms’ localization performance has decreased, as shown by the simulation results. In any considered case of measurement noise, the RMSE performances of the WLS method do not reach the CRLB. Furthermore, the SDP method performs poorly, especially when the measurement noise is high (esp. *p* > 20 dB).

**Figure 8:**
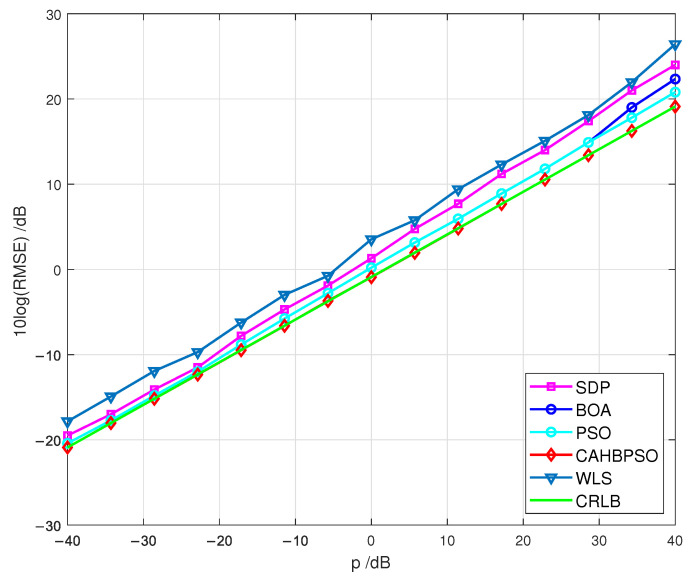
RMSEs in relation to measurement noise *p* for a third simulation scenario.

For the third simulation scenario, where the position of the passive target is randomly generated inside the examined region for each simulation run, the RMSEs of all evaluated algorithms as the function of *p* are shown and compared against the CRLB in Figure 8.

As shown in Figure 8, the proposed CAHBPSO method achieves CRLB accuracy throughout the whole range of *p* and outperforms the WLS, SDP, BOA, and PSO algorithms in terms of localization accuracy.

When comparing the numerical results of the various simulation scenarios represented in Figure 6, Figure 7, Figure 8, it can be seen that when the target is positioned inside the convex hull of the transmitters, the examined methods perform well. When the target is situated outside of the convex hull of the transmitters, the RMSE performance is higher. Furthermore, the RMSEs of the proposed CAHBPSO method achieve the CRLB throughout the whole range of *p* in every simulated scenario studied.

**Figure 9:**
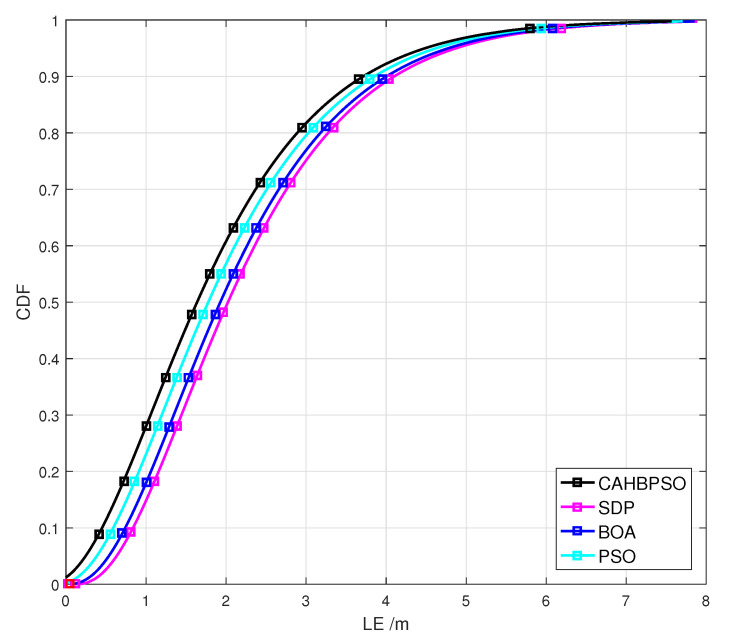
CDFs of passive target localization error of the considered algorithms for the first simulation scenario.

To further evaluate the performance of all considered algorithms, the cumulative distribution functions (CDFs) of the localization error are obtained for the variance of measurement noise $\sigma_{}^{2}=10\;m^{2}$. The localization error (LE) is defined as $LE={∥{\hat{x}}^{\left(n\right)}-x∥}_{2},\forall n\in \left\{1,\ldots ,N_{m}\right\}$. Therefore, Figure 9 shows the corresponding CDFs in terms of the localization error, for the first simulation scenario, obtained for each algorithm.

From the results in Figure 9, it is observed that in 90% of the cases, the proposed CAHBPSO algorithm provides a localization error of less than 3.66 m, while the SDP, BOA, and PSO algorithms have localization errors less than 4.1m, 4.0 m, and 3.86 m, respectively. As a result, when compared to the other algorithms studied, the suggested CAHBPSO method has the lowest localization error according to CDFs.

**Figure 10:**
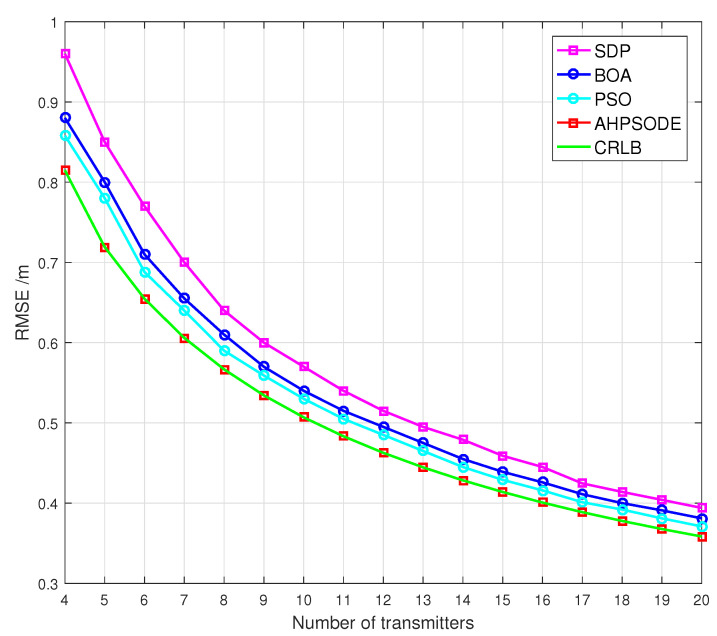
The RMSEs of the algorithms under consideration for $\sigma_{}^{2}=1\;m^{2}$, as a function of the number of transmitters.

Finally, the effect of increasing the number of transmitters on the localization accuracy is investigated for the second simulation scenario. In this respect, the *i*th transmitter is placed at the following coordinates

(56) $$x_{i}^{t}=\left[\begin{array}{c} R\cos\varphi_{i}\\ R\sin\varphi_{i} \end{array}\right],\;i\in \left\{1,2,\ldots ,N^{j}\right\}$$

where $R=80\sqrt{2}$ m is the radius of a circle, $\varphi_{i}=2\pi /N^{j}\;$ denotes the angular separation between transmitters, and $N^{j}$ is the number of transmitters taken for simulation. Hence, the RMSE performances of the considered algorithms as a function of number of transmitters are depicted in Figure 10, for the variance of measurement noise $\sigma_{}^{2}=1\;m^{2}$.

As shown in Figure 10, the RMSE performances of all considered algorithms significantly improve when the number of transmitters is increased from 4 to 10. Moreover, the proposed CAHBPSO algorithm provides 56% improvement in localization accuracy when the number of transmitters is increased from 4 to 20. Based on the simulation findings, it is determined that, when compared to other investigated methods, the performance of the proposed CAHBPSO algorithm is not susceptible to excessive measurement noise and changes in network topology.

#### Computational Complexity of the Considered Algorithms

The computational complexity of the proposed CAHBPSO and other algorithms that were taken into consideration, as well as the typical computation time required to arrive at the overall optimal solution, are analyzed and compared in this subsection. In the literature, it is shown that the computational complexity of SDP method is $O\left(16\left(N^{2}n+Nn^{2}\right)+G_{\max}{\left(n+1\right)}^{3}+4{\left(2n+3\right)}^{3.5}\right)$ [7]. Furthermore, the appropriate computational complexity of PSO algorithm can be written as $O\left(G_{\max}N_{P}\left(n+f\right)\right)$, while the complexity of BOA is $O\left(G_{\max}\left(n\times N_{P}+n\times f\right)\right)$ [70], where $\left(f\right)$ denotes the computational complexity of evaluating the considered objective function. All butterflies are sorted according to the objective function value in one generation of the proposed CAHBPSO algorithm, where the average computing complexity of this operation is $O\left(N_{P}log\left(N_{P}\right)\right)$. After sorting, the time complexity of calculating the fragrance of each butterfly is $O\left(N_{P}\right)$. Then, in the global and local search phase of the algorithm, each butterfly goes through the process of exploring the search space, where the average time complexity required to update the position of ith butterfly is $O\left(N_{P}\times n\right)+O\left(N_{P}\right)\times O\left(f\right)$. Therefore, the overall computing complexity of CAHBPSO algorithm is determined as $O\left(N_{P}log\left(N_{P}\right)\right)+O\left(N_{P}\right)+G_{\max}\left(O\left(N_{P}\times n\right)+O\left(N_{P}\right)\times O\left(f\right)\right)$, which can be simplified to $O\left(N_{P}log\left(N_{P}\right)\right)+G_{\max}\left(O\left(N_{P}\times n\right)+O\left(N_{P}\right)\times O\left(f\right)\right)$.

Following that, the average computing time required for identifying the global optimal solution is examined, as another crucial element influencing the performance of the algorithms under consideration. An identical PC with a 3.2 GHz CPU and 16 GB of RAM is used for the analysis. In this regard, for each simulation scenario the average computational times required to obtain global optimum, for each considered algorithm, are shown in Table 4.

As shown in Table 4, The BOA technique has the fastest implementation, whereas the SDP method shows the longest computing time considering all evaluated methods. Furthermore, the results reveal that the proposed CAHBPSO method provides the optimum balance of localization accuracy and average computation time to attain the global optimal solution.

## 11. Conclusions

In this paper, the passive target localization problem based on the noisy TDOA measurements, obtained from multiple transmitters and a single receiver, is considered and investigated. In this regard, a hybridization of the BOA and PSO algorithms, named CAHBPSO algorithm, is proposed to solve the considered localization problem with high accuracy, even in the presence of large measurement noise. In the proposed algorithm, an adaptive parameter is introduced to combine global search and local search phases of the BOA algorithm with the PSO algorithm, with the aim to maintain an effective balance between exploration and exploitation abilities during the optimization process. Moreover, to improve convergence and maintain population diversity, chaos theory is incorporated into the inertia weight parameter of the PSO algorithm and an adaptive strategy has been employed to update the value of the sensory fragrance of the BOA. To assess the performance of the discussed techniques, the corresponding CRLB for the passive target localization issue is derived. In addition, a statistical analysis is carried out to compare the proposed CAHBPSO algorithm optimization performance with that of well-known algorithms in the literature on a set of CEC2014 benchmark test problems.

It can be shown from the statistical comparisons between CAHBPSO and other well-known algorithms in the literature that the hybrid method suggested in this paper demonstrates better optimization performance. Furthermore, it is concluded that the proposed CAHBPSO algorithm attains the CRLB accuracy and provides better localization performance compared to the WLS, SDP, BOA, and PSO algorithms. In addition, it is observed that the CAHBPSO algorithm shows lower sensitivity to the variations in network topology and higher localization accuracy under the high measurement noise. Finally, from the analysis of the execution time and computational complexity, it is concluded that the proposed CAHBPSO algorithm provides a proper balance between localization accuracy and complexity compared to other considered algorithms.

Future studies will aim to identify the optimal network architecture for the passive target localization problem in the presence of non-line-of-sight environment. In order to improve the performance of the optimization process, research may also concentrate on performing the analysis of the sensitivity to the parameter changes.

## Figures and Tables

**Table 1:** Table presents the objective function values in terms of mean and standard deviation obtained by the considered algorithms, as a result of the numerical simulation performed on 30 CEC2014 test functions.

		CAHBPSO	SHADE	FA	BOA	PSO	HPSOBOA
		Mean (STD) Sign					
$f_{1}$	10	$2.20\times 10^{3}\left(3.48\times 10^{3}\right)$	$2.03\times 10^{6}\left(1.15\times 10^{6}\right)$+	$2.76\times 10^{8}\left(1.66\times 10^{8}\right)$+	$2.76\times 10^{6}\left(2.10\times 10^{6}\right)$+	$2.24\times 10^{6}\left(2.59\times 10^{6}\right)$+	$2.51\times 10^{8}\left(1.22\times 10^{8}\right)$+
	30	$4.31\times 10^{6}\left(4.41\times 10^{6}\right)$	$1.31\times 10^{8}\left(4.48\times 10^{7}\right)$+	$3.57\times 10^{9}\left(9.93\times 10^{8}\right)$+	$4.69\times 10^{8}\left(2.03\times 10^{8}\right)$+	$3.82\times 10^{7}\left(3.26\times 10^{7}\right)$+	$1.28\times 10^{4}\left(1.03\times 10^{4}\right)$−
	50	$7.81\times 10^{6}\left(5.49\times 10^{6}\right)$	$2.32\times 10^{8}\left(6.41\times 10^{7}\right)$+	$9.04\times 10^{9}\left(1.88\times 10^{9}\right)$+	$1.62\times 10^{9}\left(5.33\times 10^{8}\right)$+	$5.80\times 10^{7}\left(3.57\times 10^{7}\right)$+	$1.24\times 10^{10}\left(3.15\times 10^{9}\right)$+
	100	$1.53\times 10^{8}\left(6.61\times 10^{7}\right)$	$8.87\times 10^{8}\left(1.61\times 10^{8}\right)$+	$2.13\times 10^{10}\left(3.21\times 10^{9}\right)$+	$4.47\times 10^{9}\left(9.49\times 10^{8}\right)$+	$3.13\times 10^{8}\left(1.25\times 10^{8}\right)$+	$4.74\times 10^{5}\left(1.91\times 10^{5}\right)$−
$f_{2}$	10	$3.08\times 10^{2}\left(3.81\times 10^{2}\right)$	$3.04\times 10^{5}\left(1.60\times 10^{5}\right)$+	$1.28\times 10^{10}\left(4.14\times 10^{9}\right)$+	$2.78\times 10^{8}\left(2.42\times 10^{8}\right)$+	$2.69\times 10^{3}\left(3.81\times 10^{3}\right)$+	$1.22\times 10^{10}\left(2.28\times 10^{9}\right)$+
	30	$3.93\times 10^{-2}\left(2.15\times 10^{-1}\right)$	$2.84\times 10^{7}\left(9.70\times 10^{6}\right)$+	$1.34\times 10^{11}\left(1.58\times 10^{10}\right)$+	$3.76\times 10^{10}\left(5.70\times 10^{9}\right)$+	$9.43\times 10^{8}\left(8.59\times 10^{8}\right)$+	$9.79\times 10^{10}\left(1.49\times 10^{10}\right)$+
	50	$3.12\times 10^{3}\left(4.40\times 10^{3}\right)$	$2.74\times 10^{8}\left(9.07\times 10^{7}\right)$+	$2.74\times 10^{11}\left(2.32\times 10^{10}\right)$+	$9.79\times 10^{10}\left(1.49\times 10^{10}\right)$+	$5.20\times 10^{9}\left(3.36\times 10^{9}\right)$+	$1.90\times 10^{11}\left(2.41\times 10^{9}\right)$+
	100	$2.56\times 10^{6}\left(1.81\times 10^{7}\right)$	$5.52\times 10^{9}\left(1.06\times 10^{9}\right)$+	$6.44\times 10^{11}\left(4.03\times 10^{10}\right)$+	$2.36\times 10^{11}\left(1.60\times 10^{10}\right)$+	$3.18\times 10^{10}\left(7.48\times 10^{9}\right)$+	$2.31\times 10^{-12}\left(4.27\times 10^{-12}\right)$−
$f_{3}$	10	$4.01\times 10^{-3}\left(1.08\times 10^{-2}\right)$	$1.13\times 10^{3}\left(6.34\times 10^{2}\right)$+	$3.23\times 10^{5}\left(1.20\times 10^{6}\right)$+	$8.10\times 10^{2}\left(2.90\times 10^{2}\right)$+	$2.24\times 10^{3}\left(2.82\times 10^{3}\right)$+	$1.62\times 10^{4}\left(2.13\times 10^{3}\right)$+
	30	$1.44\times 10^{0}\left(1.71\times 10^{0}\right)$	$2.45\times 10^{4}\left(8.20\times 10^{3}\right)$+	$2.14\times 10^{6}\left(6.68\times 10^{6}\right)$+	$3.57\times 10^{4}\left(7.26\times 10^{3}\right)$+	$2.23\times 10^{4}\left(8.42\times 10^{3}\right)$+	$1.27\times 10^{5}\left(1.24\times 10^{4}\right)$+
	50	$2.12\times 10^{3}\left(1.96\times 10^{3}\right)$	$3.00\times 10^{5}\left(1.46\times 10^{5}\right)$+	$5.83\times 10^{5}\left(6.73\times 10^{5}\right)$+	$1.27\times 10^{5}\left(1.24\times 10^{4}\right)$+	$4.73\times 10^{4}\left(1.15\times 10^{4}\right)$+	$1.88\times 10^{5}\left(4.85\times 10^{3}\right)$+
	100	$7.25\times 10^{3}\left(3.71\times 10^{3}\right)$	$7.03\times 10^{5}\left(2.23\times 10^{5}\right)$+	$8.45\times 10^{5}\left(1.60\times 10^{5}\right)$+	$2.64\times 10^{5}\left(1.62\times 10^{4}\right)$+	$1.45\times 10^{5}\left(1.64\times 10^{4}\right)$+	$3.79\times 10^{-12}\left(5.76\times 10^{-12}\right)$−
$f_{4}$	10	$1.15\times 10^{1}\left(1.62\times 10^{1}\right)$	$3.43\times 10^{1}\left(3.25\times 10^{0}\right)$+	$2.58\times 10^{3}\left(1.25\times 10^{3}\right)$+	$6.65\times 10^{2}\left(3.59\times 10^{2}\right)$+	$3.31\times 10^{1}\left(7.85\times 10^{0}\right)$+	$2.18\times 10^{1}\left(1.63\times 10^{1}\right)$+
	30	$1.56\times 10^{2}\left(4.06\times 10^{1}\right)$	$1.64\times 10^{2}\left(2.02\times 10^{1}\right)$≈	$3.56\times 10^{4}\left(9.83\times 10^{3}\right)$+	$8.79\times 10^{3}\left(1.30\times 10^{3}\right)$+	$1.76\times 10^{2}\left(4.57\times 10^{1}\right)$−	$2.02\times 10^{0}\left(1.07\times 10^{1}\right)$−
	50	$1.78\times 10^{2}\left(3.58\times 10^{1}\right)$	$2.67\times 10^{2}\left(4.55\times 10^{1}\right)$+	$1.15\times 10^{5}\left(1.97\times 10^{4}\right)$+	$2.87\times 10^{4}\left(4.08\times 10^{3}\right)$+	$5.43\times 10^{2}\left(2.22\times 10^{2}\right)$+	$6.47\times 10^{4}\left(2.93\times 10^{3}\right)$+
	100	$5.65\times 10^{2}\left(1.18\times 10^{2}\right)$	$1.06\times 10^{3}\left(1.07\times 10^{2}\right)$+	$2.85\times 10^{5}\left(3.58\times 10^{4}\right)$+	$6.59\times 10^{4}\left(7.89\times 10^{3}\right)$+	$2.39\times 10^{3}\left(9.13\times 10^{2}\right)$+	$1.55\times 10^{2}\left(4.59\times 10^{1}\right)$−
$f_{5}$	10	$1.94\times 10^{1}\left(3.95\times 10^{0}\right)$	$2.09\times 10^{1}\left(1.41\times 10^{-1}\right)$+	$2.03\times 10^{1}\left(6.68\times 10^{-2}\right)$+	$1.99\times 10^{1}\left(1.17\times 10^{0}\right)$+	$1.98\times 10^{1}\left(2.83\times 10^{0}\right)$+	$4.37\times 10^{0}\left(7.92\times 10^{0}\right)$−
	30	$2.08\times 10^{1}\left(6.36\times 10^{-2}\right)$	$2.13\times 10^{1}\left(6.99\times 10^{-2}\right)$+	$2.10\times 10^{1}\left(4.53\times 10^{-2}\right)$+	$2.09\times 10^{1}\left(5.30\times 10^{-2}\right)$+	$2.09\times 10^{1}\left(1.08\times 10^{-1}\right)$+	$2.03\times 10^{1}\left(4.20\times 10^{-2}\right)$−
	50	$2.10\times 10^{1}\left(6.12\times 10^{-2}\right)$	$2.14\times 10^{1}\left(4.64\times 10^{-2}\right)$+	$2.12\times 10^{1}\left(3.06\times 10^{-2}\right)$+	$2.11\times 10^{1}\left(2.76\times 10^{-2}\right)$+	$2.11\times 10^{1}\left(4.71\times 10^{-2}\right)$+	$2.13\times 10^{1}\left(3.36\times 10^{-2}\right)$+
	100	$2.13\times 10^{1}\left(3.48\times 10^{-2}\right)$	$2.15\times 10^{1}\left(3.22\times 10^{-2}\right)$+	$2.13\times 10^{1}\left(2.73\times 10^{-2}\right)$+	$2.13\times 10^{1}\left(2.37\times 10^{-2}\right)$+	$2.13\times 10^{1}\left(2.50\times 10^{-2}\right)$+	$2.07\times 10^{1}\left(4.29\times 10^{-2}\right)$−
$f_{6}$	10	$7.93\times 10^{-1}\left(9.58\times 10^{-1}\right)$	$6.27\times 10^{0}\left(1.81\times 10^{0}\right)$+	$1.30\times 10^{1}\left(9.28\times 10^{-1}\right)$+	$4.55\times 10^{0}\left(4.65\times 10^{-1}\right)$+	$1.44\times 10^{0}\left(8.74\times 10^{-1}\right)$+	$3.55\times 10^{-1}\left(6.06\times 10^{-1}\right)$−
	30	$9.37\times 10^{0}\left(2.44\times 10^{0}\right)$	$3.86\times 10^{1}\left(3.11\times 10^{0}\right)$+	$4.83\times 10^{1}\left(1.42\times 10^{0}\right)$+	$2.98\times 10^{1}\left(1.43\times 10^{0}\right)$+	$1.16\times 10^{1}\left(2.34\times 10^{0}\right)$+	$2.32\times 10^{1}\left(4.19\times 10^{0}\right)$−
	50	$2.52\times 10^{1}\left(3.75\times 10^{0}\right)$	$6.80\times 10^{1}\left(5.33\times 10^{0}\right)$+	$8.41\times 10^{1}\left(1.77\times 10^{0}\right)$+	$5.89\times 10^{1}\left(2.00\times 10^{0}\right)$+	$2.65\times 10^{1}\left(3.96\times 10^{0}\right)$≈	$2.32\times 10^{1}\left(4.19\times 10^{0}\right)$−
	100	$7.52\times 10^{1}\left(5.97\times 10^{0}\right)$	$1.53\times 10^{2}\left(7.13\times 10^{0}\right)$+	$1.77\times 10^{2}\left(2.32\times 10^{0}\right)$+	$1.40\times 10^{2}\left(3.28\times 10^{0}\right)$+	$8.03\times 10^{1}\left(5.06\times 10^{0}\right)$+	$7.02\times 10^{1}\left(1.19\times 10^{1}\right)$−
$f_{7}$	10	$1.04\times 10^{-1}\left(4.43\times 10^{-2}\right)$	$9.22\times 10^{-1}\left(8.92\times 10^{-2}\right)$+	$1.96\times 10^{2}\left(4.95\times 10^{1}\right)$+	$9.20\times 10^{1}\left(3.01\times 10^{1}\right)$+	$4.78\times 10^{-1}\left(5.82\times 10^{-1}\right)$+	$7.46\times 10^{-2}\left(3.99\times 10^{-2}\right)$−
	30	$1.79\times 10^{-2}\left(2.13\times 10^{-2}\right)$	$1.22\times 10^{0}\left(6.78\times 10^{-2}\right)$+	$1.16\times 10^{3}\left(1.59\times 10^{2}\right)$+	$5.98\times 10^{2}\left(6.79\times 10^{1}\right)$+	$8.70\times 10^{0}\left(9.21\times 10^{0}\right)$+	$1.82\times 10^{-2}\left(3.05\times 10^{-2}\right)$+
	50	$1.03\times 10^{-2}\left(1.22\times 10^{-2}\right)$	$3.72\times 10^{0}\left(7.66\times 10^{-1}\right)$+	$2.68\times 10^{3}\left(2.70\times 10^{2}\right)$+	$1.27\times 10^{3}\left(8.60\times 10^{1}\right)$+	$4.60\times 10^{1}\left(3.21\times 10^{1}\right)$+	$1.82\times 10^{-2}\left(3.05\times 10^{-2}\right)$−
	100	$3.81\times 10^{-3}\left(7.32\times 10^{-3}\right)$	$5.38\times 10^{1}\left(1.27\times 10^{1}\right)$+	$5.80\times 10^{3}\left(3.77\times 10^{2}\right)$+	$2.78\times 10^{3}\left(9.78\times 10^{1}\right)$+	$2.73\times 10^{2}\left(6.99\times 10^{1}\right)$+	$1.01\times 10^{-1}\left(3.33\times 10^{-1}\right)$+
$f_{8}$	10	$5.85\times 10^{-2}\left(2.36\times 10^{-1}\right)$	$3.84\times 10^{1}\left(8.60\times 10^{0}\right)$+	$1.15\times 10^{2}\left(1.45\times 10^{1}\right)$+	$3.98\times 10^{1}\left(6.06\times 10^{0}\right)$+	$5.53\times 10^{0}\left(3.07\times 10^{0}\right)$+	$9.48\times 10^{1}\left(5.62\times 10^{0}\right)$+
	30	$1.27\times 10^{1}\left(3.29\times 10^{0}\right)$	$2.24\times 10^{2}\left(2.01\times 10^{1}\right)$+	$4.97\times 10^{2}\left(3.56\times 10^{1}\right)$+	$2.70\times 10^{2}\left(1.59\times 10^{1}\right)$+	$6.29\times 10^{1}\left(1.35\times 10^{1}\right)$+	$2.50\times 10^{1}\left(2.23\times 10^{1}\right)$−
	50	$3.86\times 10^{1}\left(6.75\times 10^{0}\right)$	$4.38\times 10^{2}\left(2.91\times 10^{1}\right)$+	$9.32\times 10^{2}\left(4.80\times 10^{1}\right)$+	$5.51\times 10^{2}\left(2.24\times 10^{1}\right)$+	$1.73\times 10^{2}\left(3.13\times 10^{1}\right)$+	$2.50\times 10^{1}\left(2.23\times 10^{1}\right)$−
	100	$1.37\times 10^{2}\left(2.47\times 10^{1}\right)$	$1.02\times 10^{3}\left(4.43\times 10^{1}\right)$+	$2.05\times 10^{3}\left(5.06\times 10^{1}\right)$+	$1.27\times 10^{3}\left(2.83\times 10^{1}\right)$+	$5.56\times 10^{2}\left(4.88\times 10^{1}\right)$+	$2.12\times 10^{2}\left(5.37\times 10^{1}\right)$+
$f_{9}$	10	$4.48\times 10^{0}\left(2.00\times 10^{0}\right)$	$5.79\times 10^{1}\left(1.05\times 10^{1}\right)$+	$1.20\times 10^{2}\left(1.37\times 10^{1}\right)$+	$3.87\times 10^{1}\left(6.38\times 10^{0}\right)$+	$1.04\times 10^{1}\left(5.22\times 10^{0}\right)$+	$4.86\times 10^{-1}\left(8.27\times 10^{-1}\right)$−
	30	$6.19\times 10^{1}\left(1.59\times 10^{1}\right)$	$2.69\times 10^{2}\left(2.42\times 10^{1}\right)$+	$6.10\times 10^{2}\left(4.43\times 10^{1}\right)$+	$2.90\times 10^{2}\left(1.80\times 10^{1}\right)$+	$8.12\times 10^{1}\left(2.19\times 10^{1}\right)$+	$8.92\times 10^{1}\left(2.34\times 10^{1}\right)$−
	50	$1.34\times 10^{2}\left(2.48\times 10^{1}\right)$	$5.02\times 10^{2}\left(3.41\times 10^{1}\right)$+	$1.16\times 10^{3}\left(6.09\times 10^{1}\right)$+	$6.10\times 10^{2}\left(2.70\times 10^{1}\right)$+	$1.82\times 10^{2}\left(3.17\times 10^{1}\right)$+	$8.92\times 10^{1}\left(2.34\times 10^{1}\right)$−
	100	$3.43\times 10^{2}\left(5.85\times 10^{1}\right)$	$1.13\times 10^{3}\left(5.30\times 10^{1}\right)$+	$2.49\times 10^{3}\left(9.12\times 10^{1}\right)$+	$1.38\times 10^{3}\left(4.42\times 10^{1}\right)$+	$5.63\times 10^{2}\left(6.26\times 10^{1}\right)$+	$3.47\times 10^{2}\left(6.33\times 10^{1}\right)$+
$f_{10}$	10	$5.69\times 10^{1}\left(6.45\times 10^{1}\right)$	$1.58\times 10^{3}\left(3.08\times 10^{2}\right)$+	$2.04\times 10^{3}\left(1.45\times 10^{2}\right)$+	$9.91\times 10^{2}\left(1.20\times 10^{2}\right)$+	$2.16\times 10^{2}\left(1.29\times 10^{2}\right)$+	$4.42\times 10^{-1}\left(9.31\times 10^{-1}\right)$−
	30	$5.05\times 10^{2}\left(2.49\times 10^{2}\right)$	$7.46\times 10^{3}\left(5.76\times 10^{2}\right)$+	$8.61\times 10^{3}\left(2.09\times 10^{2}\right)$+	$6.59\times 10^{3}\left(2.85\times 10^{2}\right)$+	$1.97\times 10^{3}\left(4.69\times 10^{2}\right)$+	$1.49\times 10^{0}\left(1.15\times 10^{0}\right)$−
	50	$1.10\times 10^{3}\left(3.56\times 10^{2}\right)$	$1.39\times 10^{4}\left(8.79\times 10^{2}\right)$+	$1.55\times 10^{4}\left(1.87\times 10^{2}\right)$+	$1.29\times 10^{4}\left(3.37\times 10^{2}\right)$+	$5.28\times 10^{3}\left(7.33\times 10^{2}\right)$+	$1.49\times 10^{0}\left(1.15\times 10^{0}\right)$−
	100	$3.96\times 10^{3}\left(7.39\times 10^{2}\right)$	$3.18\times 10^{4}\left(1.11\times 10^{3}\right)$+	$3.39\times 10^{4}\left(3.52\times 10^{2}\right)$+	$3.02\times 10^{4}\left(6.48\times 10^{2}\right)$+	$1.56\times 10^{4}\left(9.18\times 10^{2}\right)$+	$1.60\times 10^{0}\left(1.02\times 10^{0}\right)$−
$f_{11}$	10	$1.79\times 10^{2}\left(1.32\times 10^{2}\right)$	$2.14\times 10^{3}\left(2.62\times 10^{2}\right)$+	$2.07\times 10^{3}\left(9.23\times 10^{1}\right)$+	$1.07\times 10^{3}\left(1.36\times 10^{2}\right)$+	$3.26\times 10^{2}\left(1.67\times 10^{2}\right)$+	$1.30\times 10^{1}\left(1.13\times 10^{1}\right)$−
	30	$2.54\times 10^{3}\left(5.97\times 10^{2}\right)$	$9.01\times 10^{3}\left(4.31\times 10^{2}\right)$+	$8.55\times 10^{3}\left(1.95\times 10^{2}\right)$+	$6.93\times 10^{3}\left(2.24\times 10^{2}\right)$+	$2.55\times 10^{3}\left(7.53\times 10^{2}\right)$≈	$3.88\times 10^{3}\left(3.89\times 10^{2}\right)$−
	50	$5.68\times 10^{3}\left(1.22\times 10^{3}\right)$	$1.62\times 10^{4}\left(7.22\times 10^{2}\right)$+	$1.54\times 10^{4}\left(2.35\times 10^{2}\right)$+	$1.32\times 10^{4}\left(4.71\times 10^{2}\right)$+	$5.65\times 10^{3}\left(7.51\times 10^{2}\right)$≈	$3.88\times 10^{3}\left(3.89\times 10^{2}\right)$−
	100	$1.64\times 10^{4}\left(4.62\times 10^{3}\right)$	$3.47\times 10^{4}\left(9.68\times 10^{2}\right)$+	$3.36\times 10^{4}\left(3.37\times 10^{2}\right)$+	$3.07\times 10^{4}\left(6.24\times 10^{2}\right)$+	$1.38\times 10^{4}\left(2.76\times 10^{3}\right)$−	$1.23\times 10^{4}\left(1.05\times 10^{3}\right)$−
$f_{12}$	10	$1.05\times 10^{-1}\left(1.24\times 10^{-1}\right)$	$3.39\times 10^{0}\left(1.02\times 10^{0}\right)$+	$1.08\times 10^{0}\left(1.77\times 10^{-1}\right)$+	$9.85\times 10^{-1}\left(1.31\times 10^{-1}\right)$+	$3.31\times 10^{-1}\left(3.61\times 10^{-1}\right)$+	$1.12\times 10^{-1}\left(2.90\times 10^{-2}\right)$+
	30	$1.42\times 10^{0}\left(6.26\times 10^{-1}\right)$	$5.74\times 10^{0}\left(1.08\times 10^{0}\right)$+	$2.54\times 10^{0}\left(2.68\times 10^{-1}\right)$+	$2.40\times 10^{0}\left(2.40\times 10^{-1}\right)$+	$1.46\times 10^{0}\left(1.07\times 10^{0}\right)$≈	$3.08\times 10^{-1}\left(4.45\times 10^{-2}\right)$−
	50	$2.35\times 10^{0}\left(5.58\times 10^{-1}\right)$	$6.91\times 10^{0}\left(8.13\times 10^{-1}\right)$+	$3.50\times 10^{0}\left(2.46\times 10^{-1}\right)$+	$3.41\times 10^{0}\left(3.14\times 10^{-1}\right)$+	$1.82\times 10^{0}\left(1.57\times 10^{0}\right)$−	$3.08\times 10^{-1}\left(4.45\times 10^{-2}\right)$−
	100	$3.48\times 10^{0}\left(5.31\times 10^{-1}\right)$	$6.63\times 10^{0}\left(6.11\times 10^{-1}\right)$+	$4.24\times 10^{0}\left(2.40\times 10^{-1}\right)$+	$4.10\times 10^{0}\left(2.60\times 10^{-1}\right)$+	$3.35\times 10^{0}\left(1.48\times 10^{0}\right)$−	$5.84\times 10^{-1}\left(6.63\times 10^{-2}\right)$−
$f_{13}$	10	$1.06\times 10^{-1}\left(4.92\times 10^{-2}\right)$	$5.55\times 10^{-1}\left(1.42\times 10^{-1}\right)$+	$5.03\times 10^{0}\left(1.15\times 10^{0}\right)$+	$2.74\times 10^{0}\left(5.86\times 10^{-1}\right)$+	$1.13\times 10^{-1}\left(4.66\times 10^{-2}\right)$≈	$6.02\times 10^{-2}\left(1.36\times 10^{-2}\right)$−
	30	$3.69\times 10^{-1}\left(9.46\times 10^{-2}\right)$	$8.88\times 10^{-1}\left(1.76\times 10^{-1}\right)$+	$1.05\times 10^{1}\left(1.03\times 10^{0}\right)$+	$7.10\times 10^{0}\left(5.29\times 10^{-1}\right)$+	$3.21\times 10^{-1}\left(2.00\times 10^{-1}\right)$−	$3.90\times 10^{-1}\left(6.43\times 10^{-2}\right)$−
	50	$5.93\times 10^{-1}\left(9.93\times 10^{-2}\right)$	$1.09\times 10^{0}\left(1.85\times 10^{-1}\right)$+	$1.22\times 10^{1}\left(9.85\times 10^{-1}\right)$+	$7.85\times 10^{0}\left(3.08\times 10^{-1}\right)$+	$6.10\times 10^{-1}\left(2.55\times 10^{-1}\right)$≈	$3.90\times 10^{-1}\left(6.43\times 10^{-2}\right)$−
	100	$6.60\times 10^{-1}\left(7.34\times 10^{-2}\right)$	$1.15\times 10^{0}\left(1.63\times 10^{-1}\right)$+	$1.43\times 10^{1}\left(6.36\times 10^{-1}\right)$+	$9.10\times 10^{0}\left(1.84\times 10^{-1}\right)$+	$1.42\times 10^{0}\left(9.73\times 10^{-1}\right)$+	$5.28\times 10^{-1}\left(5.81\times 10^{-2}\right)$−
$f_{14}$	10	$4.94\times 10^{-2}\left(1.78\times 10^{-2}\right)$	$6.00\times 10^{-1}\left(1.53\times 10^{-1}\right)$+	$5.80\times 10^{1}\left(1.26\times 10^{1}\right)$+	$2.06\times 10^{1}\left(5.32\times 10^{0}\right)$+	$1.44\times 10^{-1}\left(1.62\times 10^{-1}\right)$+	$3.77\times 10^{-2}\left(1.37\times 10^{-2}\right)$−
	30	$2.87\times 10^{-1}\left(1.55\times 10^{-1}\right)$	$1.13\times 10^{0}\left(4.16\times 10^{-1}\right)$+	$4.23\times 10^{2}\left(5.65\times 10^{1}\right)$+	$2.23\times 10^{2}\left(2.31\times 10^{1}\right)$+	$7.61\times 10^{-1}\left(1.44\times 10^{0}\right)$+	$2.99\times 10^{-1}\left(7.19\times 10^{-2}\right)$−
	50	$5.78\times 10^{-1}\left(2.97\times 10^{-1}\right)$	$1.38\times 10^{0}\left(5.45\times 10^{-1}\right)$+	$6.91\times 10^{2}\left(6.99\times 10^{1}\right)$+	$3.20\times 10^{2}\left(2.64\times 10^{1}\right)$+	$1.03\times 10^{1}\left(9.86\times 10^{0}\right)$+	$2.99\times 10^{-1}\left(7.19\times 10^{-2}\right)$−
	100	$3.99\times 10^{-1}\left(1.77\times 10^{-1}\right)$	$4.55\times 10^{0}\left(2.57\times 10^{0}\right)$+	$1.72\times 10^{3}\left(1.19\times 10^{2}\right)$+	$8.29\times 10^{2}\left(3.69\times 10^{1}\right)$+	$8.44\times 10^{1}\left(2.16\times 10^{1}\right)$+	$3.29\times 10^{-1}\left(3.16\times 10^{-2}\right)$−
$f_{15}$	10	$7.17\times 10^{-1}\left(2.44\times 10^{-1}\right)$	$5.65\times 10^{0}\left(9.89\times 10^{-1}\right)$+	$1.87\times 10^{5}\left(1.45\times 10^{5}\right)$+	$2.01\times 10^{2}\left(2.15\times 10^{2}\right)$+	$1.00\times 10^{0}\left(4.28\times 10^{-1}\right)$+	$4.86\times 10^{4}\left(2.37\times 10^{4}\right)$+
	30	$6.15\times 10^{0}\left(1.86\times 10^{0}\right)$	$2.65\times 10^{1}\left(2.57\times 10^{0}\right)$+	$2.09\times 10^{7}\left(1.29\times 10^{7}\right)$+	$4.64\times 10^{4}\left(2.82\times 10^{4}\right)$+	$1.48\times 10^{1}\left(1.52\times 10^{1}\right)$+	$1.48\times 10^{7}\left(3.72\times 10^{6}\right)$+
	50	$1.62\times 10^{1}\left(4.85\times 10^{0}\right)$	$5.92\times 10^{1}\left(8.10\times 10^{0}\right)$+	$1.55\times 10^{8}\left(6.36\times 10^{7}\right)$+	$8.49\times 10^{5}\left(3.97\times 10^{5}\right)$+	$4.91\times 10^{2}\left(8.53\times 10^{2}\right)$+	$1.48\times 10^{7}\left(3.72\times 10^{6}\right)$+
	100	$7.03\times 10^{1}\left(1.39\times 10^{1}\right)$	$2.12\times 10^{3}\left(1.55\times 10^{3}\right)$+	$6.26\times 10^{8}\left(1.85\times 10^{8}\right)$+	$7.84\times 10^{6}\left(2.61\times 10^{6}\right)$+	$1.38\times 10^{4}\left(9.95\times 10^{3}\right)$+	$4.46\times 10^{7}\left(5.50\times 10^{6}\right)$+
$f_{16}$	10	$1.42\times 10^{0}\left(7.03\times 10^{-1}\right)$	$4.15\times 10^{0}\left(2.00\times 10^{-1}\right)$+	$3.96\times 10^{0}\left(1.45\times 10^{-1}\right)$+	$2.80\times 10^{0}\left(2.30\times 10^{-1}\right)$+	$1.90\times 10^{0}\left(5.79\times 10^{-1}\right)$+	$3.70\times 10^{0}\left(3.63\times 10^{-2}\right)$+
	30	$1.04\times 10^{1}\left(7.71\times 10^{-1}\right)$	$1.40\times 10^{1}\left(2.83\times 10^{-1}\right)$+	$1.35\times 10^{1}\left(1.50\times 10^{-1}\right)$+	$1.25\times 10^{1}\left(1.82\times 10^{-1}\right)$+	$1.03\times 10^{1}\left(7.37\times 10^{-1}\right)$≈	$2.32\times 10^{1}\left(5.31\times 10^{-2}\right)$+
	50	$2.05\times 10^{1}\left(8.38\times 10^{-1}\right)$	$2.39\times 10^{1}\left(2.54\times 10^{-1}\right)$+	$2.32\times 10^{1}\left(1.47\times 10^{-1}\right)$+	$2.22\times 10^{1}\left(1.81\times 10^{-1}\right)$+	$1.95\times 10^{1}\left(9.41\times 10^{-1}\right)$−	$2.32\times 10^{1}\left(5.31\times 10^{-2}\right)$+
	100	$4.56\times 10^{1}\left(4.68\times 10^{-1}\right)$	$4.85\times 10^{1}\left(3.34\times 10^{-1}\right)$+	$4.75\times 10^{1}\left(1.77\times 10^{-1}\right)$+	$4.64\times 10^{1}\left(2.31\times 10^{-1}\right)$+	$4.35\times 10^{1}\left(1.77\times 10^{0}\right)$−	$4.76\times 10^{1}\left(1.65\times 10^{-1}\right)$+
$f_{17}$	10	$1.12\times 10^{3}\left(1.12\times 10^{3}\right)$	$4.78\times 10^{4}\left(5.45\times 10^{4}\right)$+	$9.50\times 10^{6}\left(1.06\times 10^{7}\right)$+	$1.28\times 10^{3}\left(3.00\times 10^{2}\right)$+	$4.25\times 10^{3}\left(3.01\times 10^{3}\right)$+	$6.57\times 10^{5}\left(1.29\times 10^{5}\right)$+
	30	$3.21\times 10^{5}\left(2.55\times 10^{5}\right)$	$1.36\times 10^{7}\left(7.33\times 10^{6}\right)$+	$3.00\times 10^{8}\left(1.33\times 10^{8}\right)$+	$4.46\times 10^{6}\left(4.14\times 10^{6}\right)$+	$1.31\times 10^{6}\left(1.46\times 10^{6}\right)$+	$8.49\times 10^{8}\left(2.51\times 10^{7}\right)$+
	50	$1.17\times 10^{6}\left(9.36\times 10^{5}\right)$	$4.56\times 10^{7}\left(1.86\times 10^{7}\right)$+	$1.17\times 10^{9}\left(3.51\times 10^{8}\right)$+	$7.92\times 10^{7}\left(5.10\times 10^{7}\right)$+	$2.93\times 10^{6}\left(2.81\times 10^{6}\right)$+	$8.49\times 10^{8}\left(2.51\times 10^{7}\right)$+
	100	$1.31\times 10^{7}\left(8.27\times 10^{6}\right)$	$1.89\times 10^{8}\left(6.03\times 10^{7}\right)$+	$3.53\times 10^{9}\left(8.60\times 10^{8}\right)$+	$6.68\times 10^{8}\left(2.88\times 10^{8}\right)$+	$2.38\times 10^{7}\left(1.17\times 10^{7}\right)$+	$2.86\times 10^{9}\left(4.30\times 10^{8}\right)$+
$f_{18}$	10	$3.56\times 10^{3}\left(4.46\times 10^{3}\right)$	$1.92\times 10^{3}\left(2.70\times 10^{3}\right)$−	$1.06\times 10^{8}\left(1.15\times 10^{8}\right)$+	$4.14\times 10^{2}\left(1.99\times 10^{2}\right)$−	$4.40\times 10^{3}\left(5.30\times 10^{3}\right)$≈	$1.96\times 10^{6}\left(2.80\times 10^{6}\right)$+
	30	$5.78\times 10^{3}\left(5.96\times 10^{3}\right)$	$4.64\times 10^{6}\left(5.29\times 10^{6}\right)$+	$9.29\times 10^{9}\left(2.74\times 10^{9}\right)$+	$1.54\times 10^{8}\left(1.99\times 10^{8}\right)$+	$2.27\times 10^{6}\left(9.20\times 10^{6}\right)$+	$2.93\times 10^{10}\left(4.38\times 10^{9}\right)$+
	50	$1.06\times 10^{3}\left(1.23\times 10^{3}\right)$	$3.44\times 10^{6}\left(2.13\times 10^{6}\right)$+	$2.90\times 10^{10}\left(6.79\times 10^{9}\right)$+	$4.80\times 10^{9}\left(2.14\times 10^{9}\right)$+	$3.86\times 10^{7}\left(9.42\times 10^{7}\right)$+	$2.93\times 10^{10}\left(4.38\times 10^{9}\right)$+
	100	$1.46\times 10^{4}\left(8.49\times 10^{4}\right)$	$1.66\times 10^{7}\left(6.25\times 10^{6}\right)$+	$8.05\times 10^{10}\left(1.20\times 10^{10}\right)$+	$2.19\times 10^{10}\left(4.77\times 10^{9}\right)$+	$5.14\times 10^{8}\left(4.23\times 10^{8}\right)$+	$4.90\times 10^{10}\left(2.59\times 10^{9}\right)$+
$f_{19}$	10	$1.06\times 10^{0}\left(8.49\times 10^{-1}\right)$	$3.98\times 10^{0}\left(1.11\times 10^{0}\right)$+	$7.35\times 10^{1}\left(4.40\times 10^{1}\right)$+	$8.00\times 10^{0}\left(3.21\times 10^{0}\right)$+	$2.06\times 10^{0}\left(7.88\times 10^{-1}\right)$+	$9.37\times 10^{1}\left(3.80\times 10^{1}\right)$+
	30	$6.80\times 10^{0}\left(1.77\times 10^{0}\right)$	$1.53\times 10^{1}\left(1.88\times 10^{0}\right)$+	$1.22\times 10^{3}\left(4.46\times 10^{2}\right)$+	$3.48\times 10^{2}\left(9.00\times 10^{1}\right)$+	$2.56\times 10^{1}\left(2.02\times 10^{1}\right)$+	$5.27\times 10^{3}\left(1.16\times 10^{3}\right)$+
	50	$6.35\times 10^{1}\left(1.57\times 10^{1}\right)$	$6.74\times 10^{1}\left(9.25\times 10^{0}\right)$≈	$4.78\times 10^{3}\left(1.42\times 10^{3}\right)$+	$1.43\times 10^{3}\left(4.46\times 10^{2}\right)$+	$6.87\times 10^{1}\left(2.55\times 10^{1}\right)$≈	$5.27\times 10^{3}\left(1.16\times 10^{3}\right)$+
	100	$1.64\times 10^{2}\left(1.74\times 10^{1}\right)$	$1.83\times 10^{2}\left(1.76\times 10^{1}\right)$+	$2.14\times 10^{4}\left(3.77\times 10^{3}\right)$+	$6.46\times 10^{3}\left(1.10\times 10^{3}\right)$+	$2.92\times 10^{2}\left(6.55\times 10^{1}\right)$+	$1.55\times 10^{4}\left(7.68\times 10^{2}\right)$+
$f_{20}$	10	$3.21\times 10^{0}\left(2.73\times 10^{0}\right)$	$3.52\times 10^{1}\left(1.46\times 10^{1}\right)$+	$1.88\times 10^{6}\left(4.62\times 10^{6}\right)$+	$5.04\times 10^{2}\left(2.79\times 10^{2}\right)$+	$9.70\times 10^{2}\left(2.18\times 10^{3}\right)$+	$8.08\times 10^{4}\left(2.04\times 10^{4}\right)$+
	30	$1.30\times 10^{2}\left(5.76\times 10^{1}\right)$	$9.76\times 10^{4}\left(9.60\times 10^{4}\right)$+	$9.78\times 10^{6}\left(9.55\times 10^{6}\right)$+	$3.92\times 10^{4}\left(1.60\times 10^{4}\right)$+	$8.39\times 10^{3}\left(5.39\times 10^{3}\right)$+	$4.21\times 10^{5}\left(6.46\times 10^{4}\right)$+
	50	$9.05\times 10^{2}\left(4.29\times 10^{2}\right)$	$1.25\times 10^{6}\left(1.63\times 10^{6}\right)$+	$1.46\times 10^{7}\left(1.28\times 10^{7}\right)$+	$6.84\times 10^{4}\left(2.24\times 10^{4}\right)$+	$1.35\times 10^{4}\left(6.09\times 10^{3}\right)$+	$4.21\times 10^{5}\left(6.46\times 10^{4}\right)$+
	100	$8.85\times 10^{3}\left(2.95\times 10^{3}\right)$	$2.75\times 10^{6}\left(2.31\times 10^{6}\right)$+	$4.89\times 10^{7}\left(3.80\times 10^{7}\right)$+	$2.90\times 10^{5}\left(7.67\times 10^{4}\right)$+	$4.86\times 10^{4}\left(1.74\times 10^{4}\right)$+	$1.36\times 10^{7}\left(6.91\times 10^{6}\right)$+
$f_{21}$	10	$4.75\times 10^{1}\left(5.69\times 10^{1}\right)$	$2.10\times 10^{3}\left(6.44\times 10^{3}\right)$+	$2.04\times 10^{6}\left(2.17\times 10^{6}\right)$+	$3.56\times 10^{3}\left(1.43\times 10^{3}\right)$+	$4.26\times 10^{3}\left(3.91\times 10^{3}\right)$+	$2.25\times 10^{6}\left(1.29\times 10^{6}\right)$+
	30	$6.37\times 10^{4}\left(4.69\times 10^{4}\right)$	$4.79\times 10^{6}\left(3.27\times 10^{6}\right)$+	$1.47\times 10^{8}\left(8.24\times 10^{7}\right)$+	$4.54\times 10^{5}\left(3.22\times 10^{5}\right)$+	$3.48\times 10^{5}\left(4.60\times 10^{5}\right)$+	$3.12\times 10^{8}\left(4.04\times 10^{6}\right)$+
	50	$7.30\times 10^{5}\left(7.94\times 10^{5}\right)$	$2.81\times 10^{7}\left(1.68\times 10^{7}\right)$+	$4.74\times 10^{8}\left(2.03\times 10^{8}\right)$+	$4.34\times 10^{6}\left(2.96\times 10^{6}\right)$+	$1.82\times 10^{6}\left(1.76\times 10^{6}\right)$+	$3.12\times 10^{8}\left(4.04\times 10^{6}\right)$+
	100	$5.74\times 10^{6}\left(4.67\times 10^{6}\right)$	$1.23\times 10^{8}\left(4.39\times 10^{7}\right)$+	$1.84\times 10^{9}\left(5.25\times 10^{8}\right)$+	$1.57\times 10^{8}\left(6.71\times 10^{7}\right)$+	$9.69\times 10^{6}\left(4.68\times 10^{6}\right)$+	$8.35\times 10^{8}\left(1.43\times 10^{8}\right)$+
$f_{22}$	10	$6.25\times 10^{0}\left(9.20\times 10^{0}\right)$	$1.43\times 10^{2}\left(1.07\times 10^{2}\right)$+	$5.06\times 10^{2}\left(1.30\times 10^{2}\right)$+	$5.66\times 10^{1}\left(1.51\times 10^{1}\right)$+	$6.11\times 10^{1}\left(5.32\times 10^{1}\right)$+	$6.45\times 10^{2}\left(1.99\times 10^{2}\right)$−
	30	$2.14\times 10^{2}\left(1.41\times 10^{2}\right)$	$1.36\times 10^{3}\left(2.33\times 10^{2}\right)$+	$1.65\times 10^{4}\left(2.11\times 10^{4}\right)$+	$1.46\times 10^{3}\left(2.64\times 10^{2}\right)$+	$3.34\times 10^{2}\left(1.61\times 10^{2}\right)$+	$2.87\times 10^{6}\left(1.22\times 10^{6}\right)$+
	50	$7.56\times 10^{2}\left(2.46\times 10^{2}\right)$	$2.76\times 10^{3}\left(3.29\times 10^{2}\right)$+	$6.81\times 10^{5}\left(6.33\times 10^{5}\right)$+	$6.27\times 10^{3}\left(4.31\times 10^{3}\right)$+	$7.20\times 10^{2}\left(3.24\times 10^{2}\right)$≈	$2.87\times 10^{6}\left(1.22\times 10^{6}\right)$+
	100	$2.25\times 10^{3}\left(4.20\times 10^{2}\right)$	$6.24\times 10^{3}\left(5.61\times 10^{2}\right)$+	$2.18\times 10^{6}\left(1.11\times 10^{6}\right)$+	$2.57\times 10^{4}\left(2.61\times 10^{4}\right)$+	$1.80\times 10^{3}\left(6.15\times 10^{2}\right)$−	$5.47\times 10^{5}\left(7.45\times 10^{4}\right)$+
$f_{23}$	10	−$7.21\times 10^{3}\left(7.13\times 10^{-12}\right)$	−$7.21\times 10^{3}\left(5.60\times 10^{-3}\right)$+	−$6.94\times 10^{3}\left(1.27\times 10^{2}\right)$+	−$7.34\times 10^{3}\left(4.51\times 10^{0}\right)$−	−$7.21\times 10^{3}\left(2.83\times 10^{0}\right)$+	−$5.33\times 10^{2}\left(2.91\times 10^{-4}\right)$+
	30	−$7.22\times 10^{3}\left(1.09\times 10^{-1}\right)$	−$7.22\times 10^{3}\left(8.39\times 10^{-1}\right)$+	−$5.62\times 10^{3}\left(4.50\times 10^{2}\right)$+	−$7.34\times 10^{3}\left(9.19\times 10^{-12}\right)$−	−$7.21\times 10^{3}\left(7.46\times 10^{0}\right)$+	−$5.21\times 10^{3}\left(1.39\times 10^{-3}\right)$−
	50	−$7.19\times 10^{3}\left(4.79\times 10^{-1}\right)$	−$7.19\times 10^{3}\left(1.48\times 10^{0}\right)$+	−$3.48\times 10^{3}\left(6.79\times 10^{2}\right)$+	−$7.34\times 10^{3}\left(9.19\times 10^{-12}\right)$−	−$7.12\times 10^{3}\left(3.20\times 10^{1}\right)$+	−$5.21\times 10^{3}\left(1.39\times 10^{-3}\right)$−
	100	−$7.18\times 10^{3}\left(2.18\times 10^{0}\right)$	−$7.10\times 10^{3}\left(1.43\times 10^{1}\right)$+	$1.40\times 10^{3}\left(1.27\times 10^{3}\right)$+	−$7.34\times 10^{3}\left(9.19\times 10^{-12}\right)$−	−$6.95\times 10^{3}\left(6.80\times 10^{1}\right)$+	−$7.34\times 10^{3}\left(1.73\times 10^{-3}\right)$−
$f_{24}$	10	−$2.04\times 10^{3}\left(4.00\times 10^{0}\right)$	−$1.99\times 10^{3}\left(8.36\times 10^{0}\right)$+	−$1.91\times 10^{3}\left(1.41\times 10^{1}\right)$+	−$2.02\times 10^{3}\left(6.91\times 10^{0}\right)$+	−$2.03\times 10^{3}\left(1.32\times 10^{1}\right)$+	−$1.01\times 10^{2}\left(8.63\times 10^{-1}\right)$+
	30	−$1.92\times 10^{3}\left(5.73\times 10^{0}\right)$	−$1.91\times 10^{3}\left(3.10\times 10^{0}\right)$+	−$1.61\times 10^{3}\left(3.12\times 10^{1}\right)$+	−$1.95\times 10^{3}\left(6.89\times 10^{-13}\right)$−	−$1.95\times 10^{3}\left(2.30\times 10^{-4}\right)$−	−$9.60\times 10^{2}\left(2.03\times 10^{-3}\right)$−
	50	−$1.88\times 10^{3}\left(3.87\times 10^{0}\right)$	−$1.86\times 10^{3}\left(3.59\times 10^{0}\right)$+	−$1.24\times 10^{3}\left(5.65\times 10^{1}\right)$+	−$1.95\times 10^{3}\left(6.89\times 10^{-13}\right)$−	−$1.95\times 10^{3}\left(2.51\times 10^{-4}\right)$−	−$9.60\times 10^{2}\left(2.03\times 10^{-3}\right)$−
	100	−$1.76\times 10^{3}\left(5.60\times 10^{0}\right)$	−$1.69\times 10^{3}\left(6.59\times 10^{0}\right)$+	−$1.89\times 10^{2}\left(9.61\times 10^{1}\right)$+	−$1.95\times 10^{3}\left(6.89\times 10^{-13}\right)$−	−$1.95\times 10^{3}\left(5.76\times 10^{-4}\right)$−	−$1.95\times 10^{3}\left(3.90\times 10^{-3}\right)$−
$f_{25}$	10	−$1.43\times 10^{3}\left(4.15\times 10^{1}\right)$	−$1.40\times 10^{3}\left(8.76\times 10^{0}\right)$+	−$1.38\times 10^{3}\left(8.08\times 10^{0}\right)$+	−$1.42\times 10^{3}\left(1.50\times 10^{1}\right)$≈	−$1.41\times 10^{3}\left(2.51\times 10^{1}\right)$≈	−$1.22\times 10^{2}\left(2.78\times 10^{-2}\right)$+
	30	−$1.40\times 10^{3}\left(1.70\times 10^{0}\right)$	−$1.37\times 10^{3}\left(6.07\times 10^{0}\right)$+	−$1.19\times 10^{3}\left(6.57\times 10^{1}\right)$+	−$1.40\times 10^{3}\left(9.19\times 10^{-13}\right)$−	−$1.39\times 10^{3}\left(3.52\times 10^{0}\right)$+	−$8.62\times 10^{2}\left(3.14\times 10^{-5}\right)$−
	50	−$1.39\times 10^{3}\left(3.60\times 10^{0}\right)$	−$1.34\times 10^{3}\left(9.93\times 10^{0}\right)$+	−$8.52\times 10^{2}\left(1.04\times 10^{2}\right)$+	−$1.40\times 10^{3}\left(9.19\times 10^{-13}\right)$−	−$1.38\times 10^{3}\left(5.53\times 10^{0}\right)$+	−$8.62\times 10^{2}\left(3.14\times 10^{-5}\right)$−
	100	−$1.33\times 10^{3}\left(1.06\times 10^{1}\right)$	−$1.25\times 10^{3}\left(1.55\times 10^{1}\right)$+	$2.21\times 10^{2}\left(2.28\times 10^{2}\right)$+	−$1.40\times 10^{3}\left(9.19\times 10^{-13}\right)$−	−$1.40\times 10^{3}\left(2.50\times 10^{1}\right)$−	−$1.40\times 10^{3}\left(7.49\times 10^{-5}\right)$−
$f_{26}$	10	−$2.72\times 10^{3}\left(4.04\times 10^{-2}\right)$	−$2.72\times 10^{3}\left(1.66\times 10^{-1}\right)$+	−$2.72\times 10^{3}\left(2.06\times 10^{0}\right)$+	−$2.72\times 10^{3}\left(7.73\times 10^{-2}\right)$+	−$2.72\times 10^{3}\left(3.21\times 10^{-2}\right)$−	−$7.87\times 10^{2}\left(6.90\times 10^{-1}\right)$−
	30	−$2.72\times 10^{3}\left(3.61\times 10^{1}\right)$	−$2.72\times 10^{3}\left(1.49\times 10^{1}\right)$+	−$2.47\times 10^{3}\left(1.23\times 10^{2}\right)$+	−$2.71\times 10^{3}\left(2.08\times 10^{1}\right)$+	−$2.70\times 10^{3}\left(4.27\times 10^{1}\right)$≈	−$2.78\times 10^{3}\left(2.42\times 10^{1}\right)$+
	50	−$2.65\times 10^{3}\left(8.53\times 10^{1}\right)$	−$2.65\times 10^{3}\left(6.42\times 10^{1}\right)$−	−$2.11\times 10^{3}\left(1.77\times 10^{2}\right)$+	−$2.64\times 10^{3}\left(3.19\times 10^{1}\right)$−	−$2.64\times 10^{3}\left(4.85\times 10^{1}\right)$≈	−$2.78\times 10^{3}\left(2.42\times 10^{1}\right)$+
	100	−$2.61\times 10^{3}\left(5.90\times 10^{1}\right)$	−$2.58\times 10^{3}\left(1.50\times 10^{1}\right)$+	−$1.19\times 10^{3}\left(2.11\times 10^{2}\right)$+	−$2.62\times 10^{3}\left(2.30\times 10^{-12}\right)$−	−$2.62\times 10^{3}\left(2.02\times 10^{-12}\right)$−	−$2.62\times 10^{3}\left(2.39\times 10^{-7}\right)$−
$f_{27}$	10	−$1.69\times 10^{4}\left(1.70\times 10^{2}\right)$	−$1.69\times 10^{4}\left(1.96\times 10^{2}\right)$≈	−$1.65\times 10^{4}\left(1.18\times 10^{2}\right)$+	−$1.72\times 10^{4}\left(2.52\times 10^{0}\right)$−	−$1.69\times 10^{4}\left(1.53\times 10^{2}\right)$≈	−$3.18\times 10^{3}\left(2.23\times 10^{-2}\right)$−
	30	−$1.67\times 10^{4}\left(9.99\times 10^{1}\right)$	−$1.64\times 10^{4}\left(1.77\times 10^{2}\right)$+	−$1.53\times 10^{4}\left(1.58\times 10^{2}\right)$+	−$1.67\times 10^{4}\left(1.57\times 10^{1}\right)$−	−$1.66\times 10^{4}\left(1.07\times 10^{2}\right)$−	−$1.10\times 10^{4}\left(2.59\times 10^{-2}\right)$−
	50	−$1.62\times 10^{4}\left(1.07\times 10^{2}\right)$	−$1.56\times 10^{4}\left(1.43\times 10^{2}\right)$+	−$1.39\times 10^{4}\left(3.77\times 10^{2}\right)$+	−$1.65\times 10^{4}\left(1.11\times 10^{2}\right)$−	−$1.62\times 10^{4}\left(9.68\times 10^{1}\right)$−	−$1.10\times 10^{4}\left(2.59\times 10^{-2}\right)$−
	100	−$1.49\times 10^{4}\left(1.80\times 10^{2}\right)$	−$1.41\times 10^{4}\left(2.32\times 10^{2}\right)$+	−$9.71\times 10^{3}\left(1.03\times 10^{3}\right)$+	−$1.50\times 10^{4}\left(5.29\times 10^{2}\right)$−	−$1.49\times 10^{4}\left(1.56\times 10^{2}\right)$≈	−$1.70\times 10^{4}\left(7.29\times 10^{-2}\right)$−
$f_{28}$	10	−$5.79\times 10^{4}\left(5.90\times 10^{1}\right)$	−$5.79\times 10^{4}\left(8.59\times 10^{1}\right)$≈	−$5.67\times 10^{4}\left(2.65\times 10^{2}\right)$+	−$5.79\times 10^{4}\left(6.97\times 10^{1}\right)$≈	−$5.79\times 10^{4}\left(7.17\times 10^{1}\right)$≈	−$6.26\times 10^{3}\left(3.34\times 10^{-2}\right)$−
	30	−$5.73\times 10^{4}\left(2.82\times 10^{2}\right)$	−$5.71\times 10^{4}\left(8.32\times 10^{1}\right)$+	−$5.02\times 10^{4}\left(7.77\times 10^{2}\right)$+	−$5.70\times 10^{4}\left(1.43\times 10^{2}\right)$+	−$5.74\times 10^{4}\left(1.43\times 10^{2}\right)$−	−$2.43\times 10^{4}\left(9.75\times 10^{-2}\right)$−
	50	−$5.65\times 10^{4}\left(4.44\times 10^{2}\right)$	−$5.59\times 10^{4}\left(1.00\times 10^{3}\right)$+	−$4.17\times 10^{4}\left(1.52\times 10^{3}\right)$+	−$5.52\times 10^{4}\left(5.24\times 10^{2}\right)$+	−$5.65\times 10^{4}\left(4.68\times 10^{2}\right)$≈	−$2.43\times 10^{4}\left(9.75\times 10^{-2}\right)$−
	100	−$5.32\times 10^{4}\left(1.21\times 10^{3}\right)$	−$5.00\times 10^{4}\left(2.77\times 10^{3}\right)$+	−$1.79\times 10^{4}\left(2.21\times 10^{3}\right)$+	−$4.84\times 10^{4}\left(1.62\times 10^{3}\right)$+	−$5.21\times 10^{4}\left(9.93\times 10^{2}\right)$+	−$5.82\times 10^{4}\left(1.43\times 10^{-1}\right)$−
$f_{29}$	10	−$2.71\times 10^{10}\left(5.97\times 10^{5}\right)$	−$2.71\times 10^{10}\left(2.84\times 10^{4}\right)$+	−$2.71\times 10^{10}\left(1.98\times 10^{7}\right)$+	−$2.71\times 10^{10}\left(5.55\times 10^{2}\right)$≈	−$2.71\times 10^{10}\left(4.15\times 10^{5}\right)$≈	−$1.02\times 10^{9}\left(8.96\times 10^{3}\right)$+
	30	−$2.70\times 10^{10}\left(3.03\times 10^{2}\right)$	−$2.70\times 10^{10}\left(8.16\times 10^{4}\right)$+	−$2.60\times 10^{10}\left(2.33\times 10^{8}\right)$+	−$2.70\times 10^{10}\left(0.00\times 10^{0}\right)$−	−$2.70\times 10^{10}\left(4.07\times 10^{5}\right)$+	−$7.37\times 10^{9}\left(1.15\times 10^{5}\right)$+
	50	−$2.70\times 10^{10}\left(1.54\times 10^{7}\right)$	−$2.70\times 10^{10}\left(5.95\times 10^{5}\right)$+	−$2.40\times 10^{10}\left(4.96\times 10^{8}\right)$+	−$2.70\times 10^{10}\left(0.00\times 10^{0}\right)$−	−$2.70\times 10^{10}\left(2.92\times 10^{6}\right)$+	−$7.37\times 10^{9}\left(1.15\times 10^{5}\right)$+
	100	−$2.71\times 10^{10}\left(1.05\times 10^{3}\right)$	−$2.71\times 10^{10}\left(1.92\times 10^{6}\right)$+	−$1.82\times 10^{10}\left(1.13\times 10^{9}\right)$+	−$2.71\times 10^{10}\left(0.00\times 10^{0}\right)$−	−$2.70\times 10^{10}\left(2.40\times 10^{7}\right)$+	−$2.71\times 10^{10}\left(1.27\times 10^{5}\right)$+
$f_{30}$	10	−$3.02\times 10^{9}\left(1.73\times 10^{2}\right)$	−$3.02\times 10^{9}\left(4.99\times 10^{2}\right)$+	−$3.02\times 10^{9}\left(1.91\times 10^{5}\right)$+	−$3.02\times 10^{9}\left(2.77\times 10^{2}\right)$+	−$3.02\times 10^{9}\left(4.53\times 10^{2}\right)$+	−$4.40\times 10^{8}\left(3.37\times 10^{4}\right)$+
	30	−$3.00\times 10^{9}\left(1.15\times 10^{3}\right)$	−$3.00\times 10^{9}\left(1.48\times 10^{4}\right)$+	−$3.00\times 10^{9}\left(4.25\times 10^{6}\right)$+	−$3.00\times 10^{9}\left(9.63\times 10^{-7}\right)$−	−$3.00\times 10^{9}\left(1.72\times 10^{4}\right)$+	−$6.15\times 10^{8}\left(3.91\times 10^{3}\right)$−
	50	−$3.00\times 10^{9}\left(4.67\times 10^{3}\right)$	−$3.00\times 10^{9}\left(7.08\times 10^{4}\right)$+	−$3.00\times 10^{9}\left(1.68\times 10^{7}\right)$+	−$3.00\times 10^{9}\left(9.63\times 10^{-7}\right)$−	−$3.00\times 10^{9}\left(4.38\times 10^{4}\right)$+	−$6.15\times 10^{8}\left(3.91\times 10^{3}\right)$−
	100	−$3.02\times 10^{9}\left(3.52\times 10^{4}\right)$	−$3.02\times 10^{9}\left(1.23\times 10^{6}\right)$+	−$2.51\times 10^{9}\left(1.44\times 10^{8}\right)$+	−$3.02\times 10^{9}\left(9.63\times 10^{-7}\right)$−	−$3.02\times 10^{9}\left(9.39\times 10^{5}\right)$+	−$3.02\times 10^{9}\left(2.04\times 10^{4}\right)$−

**Table 2:** Wilcoxon test results for D=10,30,50, and 100 obtained using CAHBPSO and other evaluated algorithms on a set of CEC2014 benchmark problems.

*D*	Algorithms	$R^{+}$	$R^{-}$	*p* Value	+	≈	−	Dec.
10	CAHBPSO versus SHADE	392	73	$6.08\times 10^{-4}$	27	2	1	+
	CAHBPSO versus FA	465	0	$1.86\times 10^{-9}$	30	0	0	+
	CAHBPSO versus BOA	376	89	$2.37\times 10^{-3}$	24	3	3	+
	CAHBPSO versus PSO	419	46	$3.45\times 10^{-5}$	23	6	1	+
	CAHBPSO versus HPSOBOA	330	153	$4.49\times 10^{-2}$	18	12	0	+
30	CAHBPSO versus SHADE	457	8	$4.66\times 10^{-8}$	29	1	0	+
	CAHBPSO versus FA	465	0	$1.86\times 10^{-9}$	30	0	0	+
	CAHBPSO versus BOA	396	69	$4.18\times 10^{-4}$	24	0	6	+
	CAHBPSO versus PSO	424	41	$1.82\times 10^{-5}$	22	5	3	+
	CAHBPSO versus HPSOBOA	234	231	$9.84\times 10^{-1}$	13	17	0	≈
50	CAHBPSO versus SHADE	441	24	$1.42\times 10^{-6}$	29	1	0	+
	CAHBPSO versus FA	465	0	$1.86\times 10^{-9}$	30	0	0	+
	CAHBPSO versus BOA	387	78	$9.52\times 10^{-4}$	24	0	6	+
	CAHBPSO versus PSO	391	74	$6.67\times 10^{-4}$	20	7	3	+
	CAHBPSO versus HPSOBOA	184	181	$3.28\times 10^{-1}$	15	15	0	≈
100	CAHBPSO versus SHADE	465	0	$1.86\times 10^{-9}$	30	0	0	+
	CAHBPSO versus FA	465	0	$1.86\times 10^{-9}$	30	0	0	+
	CAHBPSO versus BOA	389	76	$7.98\times 10^{-4}$	23	1	6	+
	CAHBPSO versus PSO	398	67	$3.45\times 10^{-4}$	22	1	7	+
	CAHBPSO versus HPSOBOA	238	227	$9.19\times 10^{-1}$	13	17	0	≈

**Table 3:** The obtained average ranks using Friedman test for all investigated algorithms across all functions and dimensions using CEC2014, with α=0.05.

Algorithm	10D	30D	50D	100D	Mean Ranking	Rank
CAHBPSO	**1.77**	**2.07**	**1.97**	**2.07**	1.97	1
HPSOBOA	3.40	2.57	3.00	2.47	2.86	2
PSO	2.97	2.97	2.87	3.00	2.95	3
BOA	3.30	3.60	3.50	3.57	3.49	4
SHADE	3.83	4.03	3.97	4.07	3.98	5
FA	5.73	5.77	5.70	5.83	5.76	6
Friedman *p* value	$3.36\times 10^{-14}$	$1.44\times 10^{-14}$	$1.58\times 10^{-13}$	$1.84\times 10^{-15}$		

**Table 4:** The computation times on average for the methods under consideration.

	SDP	PSO	BOA	CAHBPSO
Scenario 1	10.03	$3.02\times 10^{-2}$	$1.46\times 10^{-2}$	$2.06\times 10^{-1}$
Scenario 2	9.96	$1.03\times 10^{-2}$	$1.13\times 10^{-2}$	$2.07\times 10^{-1}$
Scenario 3	14.55	$1.17\times 10^{-2}$	$1.46\times 10^{-2}$	$2.48\times 10^{-1}$

## Data Availability

No new data were created or analyzed in this study. Data sharing is not applicable to this article.

## Appendix A

The logarithm of the likelihood function, which is defined in Equation (10), has the following form

(A1) $$\ln f\left(\tilde{r}\left|x\right.\right)=c-\frac{1}{2\sigma^{2}}\left({\left(\tilde{r}-Hg\left(x\right)\right)}^{T}\left(\tilde{r}-Hg\left(x\right)\right)\right),$$

From Equation (A1), the log-likelihood function’s first partial derivative with respect to x is given as

(A2) $$\frac{\partial \ln\left(f\left(\tilde{r}\left|x\right.\right)\right)}{\partial x}=-\frac{1}{2\sigma^{2}}\frac{\partial }{\partial x}\left({\left(\tilde{r}-Hg\left(x\right)\right)}^{T}\left(\tilde{r}-Hg\left(x\right)\right)\right),$$

where

(A3) $$\frac{\partial }{\partial x}\left({\left(\tilde{r}-Hg\left(x\right)\right)}^{T}\left(\tilde{r}-Hg\left(x\right)\right)\right)=-2\frac{\partial g{\left(x\right)}^{T}}{\partial x}H^{T}\left(\tilde{r}-Hg\left(x\right)\right).$$

Then, substituting Equation (A3) into Equation (A2), the following is obtained

(A4) $$\frac{\partial \ln\left(f\left(\tilde{r}\left|\;\;\;x\;\;\;\right.\right)\right)}{\partial x}=\frac{1}{\sigma^{2}}\frac{\partial g{\left(x\right)}^{T}}{\partial x}H^{T}\left(\tilde{r}-Hg\left(x\right)\right)$$

As a result, taking the partial derivative with regard to *x* the following is obtained

(A5) $$\left(\frac{\partial \ln\left(f\left(\tilde{r}\left|x\right.\right)\right)}{\partial x}\right)=\frac{1}{\sigma^{2}}{\left(\frac{\partial g\left(x\right)}{\partial x}\right)}^{T}H^{T}\left(\tilde{r}-Hg\left(x\right)\right).$$

Using the above expressions, the first element on the diagonal of the FIM is obtained as follows

(A6) $$\begin{array}{c} \begin{array}{cc} \; & F_{11}=E\left[\left(\frac{\partial \ln\left(f\left(\tilde{r}\left|x\right.\right)\right)}{\partial x}\right){\left(\frac{\partial \ln\left(f\left(\tilde{r}\left|x\right.\right)\right)}{\partial x}\right)}^{T}\right]\\ \; & =E\left[\frac{1}{\sigma^{4}}{\left(\frac{\partial g\left(x\right)}{\partial x}\right)}^{T}H^{T}\left(\tilde{r}-Hg\left(x\right)\right){\left(\tilde{r}-Hg\left(x\right)\right)}^{T}H\left(\frac{\partial g\left(x\right)}{\partial x}\right)\right]\\ \; & =\frac{1}{\sigma^{4}}{\left(\frac{\partial g\left(x\right)}{\partial x}\right)}^{T}H^{T}E\left[\left(\tilde{r}-Hg\left(x\right)\right){\left(\tilde{r}-Hg\left(x\right)\right)}^{T}\right]H\left(\frac{\partial g\left(x\right)}{\partial x}\right)\\ \; & =\frac{1}{\sigma^{2}}{\left(\frac{\partial g\left(x\right)}{\partial x}\right)}^{T}H^{T}H\left(\frac{\partial g\left(x\right)}{\partial x}\right). \end{array} \end{array}$$

The rest of the element of the FIM matrix are obtained in the same way as above, e.g.,

(A7) $$\begin{array}{c} \begin{array}{cc} \; & F_{12}=F_{21}=E\left[\left(\frac{\partial \ln\left(f\left(\tilde{r}\left|x\right.\right)\right)}{\partial x}\right){\left(\frac{\partial \ln\left(f\left(\tilde{r}\left|x\right.\right)\right)}{\partial y}\right)}^{T}\right]\\ \; & =\frac{1}{\sigma^{2}}{\left(\frac{\partial g\left(x\right)}{\partial x}\right)}^{T}H^{T}H\left(\frac{\partial g\left(x\right)}{\partial y}\right), \end{array} \end{array}$$

(A8) $$\begin{array}{c} \begin{array}{cc} \; & F_{22}=E\left[\left(\frac{\partial \ln\left(f\left(\tilde{r}\left|x\right.\right)\right)}{\partial y}\right){\left(\frac{\partial \ln\left(f\left(\tilde{r}\left|x\right.\right)\right)}{\partial y}\right)}^{T}\right]\\ \; & =\frac{1}{\sigma^{2}}{\left(\frac{\partial g\left(x\right)}{\partial y}\right)}^{T}H^{T}H\left(\frac{\partial g\left(x\right)}{\partial y}\right). \end{array} \end{array}$$

The partial derivative of $g\left(x\right)$ with respect to the components of *x* may be represented as according to Equation (7), as follows

(A9) $$\frac{\partial g(x)}{\partial x}=\left[\begin{array}{cc} \frac{x-x_{r}}{{∥x-x_{r}∥}_{2}} & \frac{y-y_{r}}{{∥x-x_{r}∥}_{2}}\\ \frac{x-x_{1}^{t}}{{∥x-x_{1}^{t}∥}_{2}} & \frac{y-y_{1}^{t}}{{∥x-x_{1}^{t}∥}_{2}}\\ \vdots & \vdots \\ \frac{x-x_{N}^{t}}{{∥x-x_{N}^{t}∥}_{2}} & \frac{y-y_{N}^{t}}{{∥x-x_{N}^{t}∥}_{2}} \end{array}\right],$$

where ∂g(x)/∂x is the N×2 Jacobian matrix. The next expression may be readily produced by doing matrix multiplication

(A10) $$\begin{array}{c} \begin{array}{cc} \; & F_{11}=\frac{1}{\sigma^{2}}{\left(\frac{\partial g(x)}{\partial x}\right)}^{T}H^{T}H\left(\frac{\partial g(x)}{\partial x}\right)\\ \; & =\frac{1}{\sigma^{2}}\sum_{i=1}^{N}{\left(\frac{x-x_{r}}{{∥x-x_{r}∥}_{2}}+\frac{x-x_{i}^{t}}{{∥x-x_{i}^{t}∥}_{2}}\right)}^{2} \end{array} \end{array}$$

The equations for the remaining elements of FIM are produced in the same way

(A11) $$\begin{array}{c} \begin{array}{cc} \; & F_{12}=F_{21}=\frac{1}{\sigma^{2}}{\left(\frac{\partial g(x)}{\partial x}\right)}^{T}H^{T}H\left(\frac{\partial g(x)}{\partial y}\right)\\ \; & =\frac{1}{\sigma^{2}}\sum_{i=1}^{N}\left(\frac{x-x_{r}}{{∥x-x_{r}∥}_{2}}+\frac{x-x_{i}^{t}}{{∥x-x_{i}^{t}∥}_{2}}\right)\left(\frac{y-y_{r}}{{∥x-x_{r}∥}_{2}}+\frac{y-y_{i}^{t}}{{∥x-x_{i}^{t}∥}_{2}}\right) \end{array} \end{array}$$

(A12) $$\begin{array}{c} \begin{array}{cc} \; & F_{22}=\frac{1}{\sigma^{2}}{\left(\frac{\partial g(x)}{\partial y}\right)}^{T}H^{T}H\left(\frac{\partial g(x)}{\partial y}\right)\\ \; & =\frac{1}{\sigma^{2}}\sum_{i=1}^{N}{\left(\frac{y-y_{r}}{{∥x-x_{r}∥}_{2}}+\frac{y-y_{i}^{t}}{{∥x-x_{i}^{t}∥}_{2}}\right)}^{2} \end{array} \end{array}$$

Finally, Equations (51)–(53) of the corresponding elements of FIM are obtained.
